# pADM patch augmentation for rotator cuff repair: *In vivo* safety and efficacy in a canine model

**DOI:** 10.3389/fbioe.2026.1912857

**Published:** 2026-10-08

**Authors:** Jianbo Li, Qingjun kong, Qian Xu, Kuansong Wang

**Affiliations:** 1 Department of Pathology, Xiangya Hospital, School of Basic Medical Science, Central South University, Changsha, Hunan, China; 2 R&D Center, BioPic (Chongqing) Biotechnology Co., Ltd., Chongqing, China

**Keywords:** acellular dermal matrix, biological patch, biomechanics, in vivo degradation, rotator cuff injury, tendon-bone healing

## Abstract

**Objective:**

Rotator cuff repair remains challenged by a high postoperative retear rate, particularly in moderate-to-large tears and in patients with poor tendon quality, where conventional suture-anchor fixation often fails to provide sufficient mechanical support during the healing process. This study aimed to systematically evaluate the *in vivo* safety, degradation characteristics, and reparative efficacy of a porcine-derived acellular dermal matrix (pADM) patch for rotator cuff augmentation in a large animal model.

**Methods:**

Bilateral infraspinatus tendon insertion injury models were established in 24 adult beagle dogs. Animals in the test group received pADM patch augmentation in combination with suture-anchor fixation, whereas those in the control group underwent conventional anchor repair alone. Comprehensive assessments including hematological analysis, gross observation, CT/MRI images, histopathology (HE and Masson staining), and biomechanical testing were performed at 2, 4, 6, and 13 weeks postoperatively.

**Results:**

Implantation of the pADM patch elicited no abnormal inflammatory or immune responses, indicating favorable biocompatibility. Imaging and histology analyses demonstrated superior tendon structural restoration and enhanced tendon-bone integration in the test group. The patch underwent progressive degradation and achieved effective host-tissue integration. Biomechanical testing revealed significantly higher maximum failure loads in the test group at all time points, indicating superior mechanical integrity of the repaired tendon (P < 0.05).

**Conclusion:**

The pADM patch exhibits favorable biosafety and biocompatibility, promotes tendon structure recovery and tendon-bone healing, and enhances biomechanical strength. Its degradation kinetics match the host tissue remodeling process, with complete resorption by 13 weeks. This acellular dermal matrix patch represents a safe and effective augmentation material for rotator cuff repair, with promising potential for clinical translation.

## 1 Introduction

Rotator cuff injury is one of the most prevalent musculoskeletal disorders of the shoulder and represents a major cause of pain, functional limitation, and reduced quality of life (Oh et al., 2018). Its pathogenesis is multifactorial and involves acute trauma, chronic tendon degeneration, repetitive mechanical overload, and age-related tissue deterioration (Oh et al., 2018; Derwin et al., 2010). Although arthroscopic anchor repair has become the standard treatment, for moderate-to-severe tears, poor tissue quality, or degenerative injuries, simple anchor repair often fails to provide adequate mechanical stability and results in slow tendon-bone healing, with postoperative retear rates as high as 20–90% (Derwin et al., 2010; Rhee and Oh, 2017; Cooper and Mirzayan, 2016). Therefore, improving the mechanical stability of the repair construct while simultaneously promoting biologically effective tendon regeneration has become a critical challenge in rotator cuff surgery (Jiang et al., 2024).

To address these limitations, patch augmentation strategies have been increasingly introduced into rotator cuff repair. In addition to providing mechanical reinforcement and reducing local stress concentration, an ideal augmentation material should also actively regulate the local healing microenvironment by promoting host cell infiltration, collagen deposition, angiogenesis, and extracellular matrix (ECM) remodeling. Various biomaterials have been explored for rotator cuff augmentation, including autologous grafts, synthetic scaffolds, and ECM-derived biological patches (Derwin et al., 2010; Jiang et al., 2024). Studies have shown that autografts (e.g., long head of biceps tendon) exhibit good biocompatibility and histocompatibility but are limited by donor-site morbidity and availability (Rhee and Oh, 2017). Allogeneic or xenogeneic patches (e.g., porcine small intestinal submucosa, SIS) possess some biological activity, but some studies report that they may elicit strong inflammatory reactions and even lead to repair failure (Walton et al., 2007). Consequently, biological patches capable of mechanical support and biological regulation have emerged as a major focus in tendon tissue engineering and regenerative repair.

Among these biomaterials, acellular dermal matrix (ADM) has emerged as a particularly promising candidate for soft-tissue augmentation due to its unique structural and biological characteristics. As a decellularized ECM-derived scaffold, ADM preserves the native three-dimensional collagen architecture, bioactive adhesion motifs, and matrix-associated signaling molecules while largely eliminating immunogenic cellular constituents (Chen et al., 2022). These properties enable ADM to support cell migration, vascular ingrowth, collagen remodeling, and constructive tissue regeneration (Chen et al., 2022; Bush and Gertzman, 2016; Mohamma et al., 2023). In particular, porcine-derived ADM (pADM) provides abundant availability, favorable mechanical properties, and controllable biodegradation, making it suitable for rotator cuff augmentation.

Despite these promising features, reports on xenogeneic ADM in rotator cuff repair remain scarce, and most existing studies-even those in large animal models-have been largely confined to short-term histological observations or isolated biomechanical measurements, without systematic elucidation of *in vivo* degradation kinetics, tissue integration pattern, or mechanical augmentation mechanism over an extended period (Cooper and Mirzayan, 2016; Adams et al., 2006). In addition, the use of autologous or allogeneic dermal matrices, which dominate the current literature, is constrained by donor availability, potential secondary morbidity, and batch-to-batch variability. These gaps highlight the need for a comprehensive, long-term evaluation of xenogeneic pADM in a clinically relevant large animal model. Therefore, the present study established a standardized rotator cuff repair model using infraspinatus tendon insertion injury in beagle dogs. Adopting a self-controlled design and integrating CT/MRI imaging, quantitative histopathology, *in vivo* degradation analysis, and biomechanical testing within a single experimental framework, we performed a multi-dimensional, multi-time-point comprehensive evaluation (at 2, 4, 6, and 13 weeks) to systematically elucidate the safety, degradation kinetics, tissue integration pattern, and mechanical augmentation mechanism of the pADM patch.

## 2 Materials and methods

### 2.1 Experimental materials

The biological pADM patch was used a (BP-01, 50 mm × 50 mm, provided by BioPic (Chongqing) Biotechnology Co., Ltd., China). The patch was manufactured through a series of decellularization, purification, and sterilization procedures designed to preserve the native extracellular matrix architecture while minimising immunogenicity. The pADM patch was systematically characterized *in vitro* prior to *in vivo* implantation, including decellularization efficiency (residual DNA and total protein content), mechanical properties, cytocompatibility, and *in vitro* degradation kinetics. Suture anchors were used as control repair materials.

Twenty-four adult beagle dogs (ordinary grade, male:female = 1:1, body weight range 13–15 kg) were supplied by Hunan Hengxinwang Biotechnology Co., Ltd. All animal protocols were approved by the Institutional Animal Care and Use Committee (IACUC). This study was conducted in strict accordance with the ARRIVE 2.0 guidelines for the design, implementation, and reporting of animal experiments (Supplementary Table S1). Animal care and experimental procedures complied with relevant guidelines for laboratory animal care, and the protocol was approved by the Institutional Animal Care and Use Committee (EP-I-D-250205-00-01).

### 2.2 Experimental design and grouping

A self-controlled design was employed. After a 7-day quarantine period, animals were randomly assigned to groups. The left shoulder joint of each dog was assigned to the test group (pADM patch combined with suture anchor repair), and the right shoulder joint served as the control group (suture anchor repair alone). Based on observation time points, animals were divided into 2W, 4W, 6W, and 13W groups (n = 6 per group). At all time points the examinations were performed under propofol anesthesia to ensure animal welfare.

### 2.3 Animal model preparation and surgical procedure

Dogs were fasted for 8 h before surgery. Aanesthesia was induced with intravenous propofol (10 mg/mL, 1 mL/kg) and maintained with isoflurane (2.0%–2.5% in oxygen), with continuous monitoring of respiration and heart rate. Standard skin preparation, disinfection, and draping were performed. An incision was made along the inferior border of the scapular spine; the skin and subcutaneous tissue were incised sequentially, and the muscle layer was bluntly dissected to expose the insertion site of the infraspinatus tendon on the humeral greater tuberosity.

The infraspinatus tendon was transected at its insertion, and a segment of approximately 5 mm × 5 mm was excised to simulate a clinical moderate-sized tear (Figure 1). Soft tissue at the footprint area was debrided, bone tunnels were prepared using a tap, and one medial-row anchor (3.5 × 16 mm) and two lateral-row anchors (3.4 × 14 mm) were sequentially implanted. In the test group, the biological patch was rehydrated with normal saline, trimmed to a final size of approximately 18 mm × 10 mm (with its native thickness preserved), placed over the tendon-bone interface, and fixed to the lateral aspect of the greater tuberosity using a suture-bridge technique. In the control group, tendon suture and anchor fixation were performed using the same anchor types and suture-bridge technique, but without patch implantation. All surgical procedures were carried out by the same experienced surgeon. The incision was closed in layers. Animals were placed in a recovery room after surgery and returned to their housing after awakening. Postoperatively, meloxicam (0.2 mL/kg on day 1, followed by 0.1 mL/kg daily, subcutaneous) was administered for 5 days for analgesia, and ceftiofur sodium (2.2 mg/kg, intramuscular) for 5 days for antibiotic prophylaxis. At the designated time points (2, 4, 6, and 13 weeks postoperatively), animals were anesthetized by intramuscular injection of Zoletil at 2.5 mg/kg, followed by intravenous administration of propofol at 3 mg/kg. After confirming the absence of corneal and pedal withdrawal reflexes, euthanasia was performed under deep anesthesia by exsanguination. Briefly, a 10 cm transverse incision was made in the femoral triangle, and the femoral artery and vein were completely severed. A wet gauze was used to continuously wipe away blood and clots from the incision site, while running tap water was applied to maintain patency of the femoral arterial opening. Death occurred within 3–5 min as a result of acute hemorrhagic shock, and was further confirmed by the absence of a heartbeat on auscultation and pupillary dilation unresponsive to light.

**FIGURE 1:**
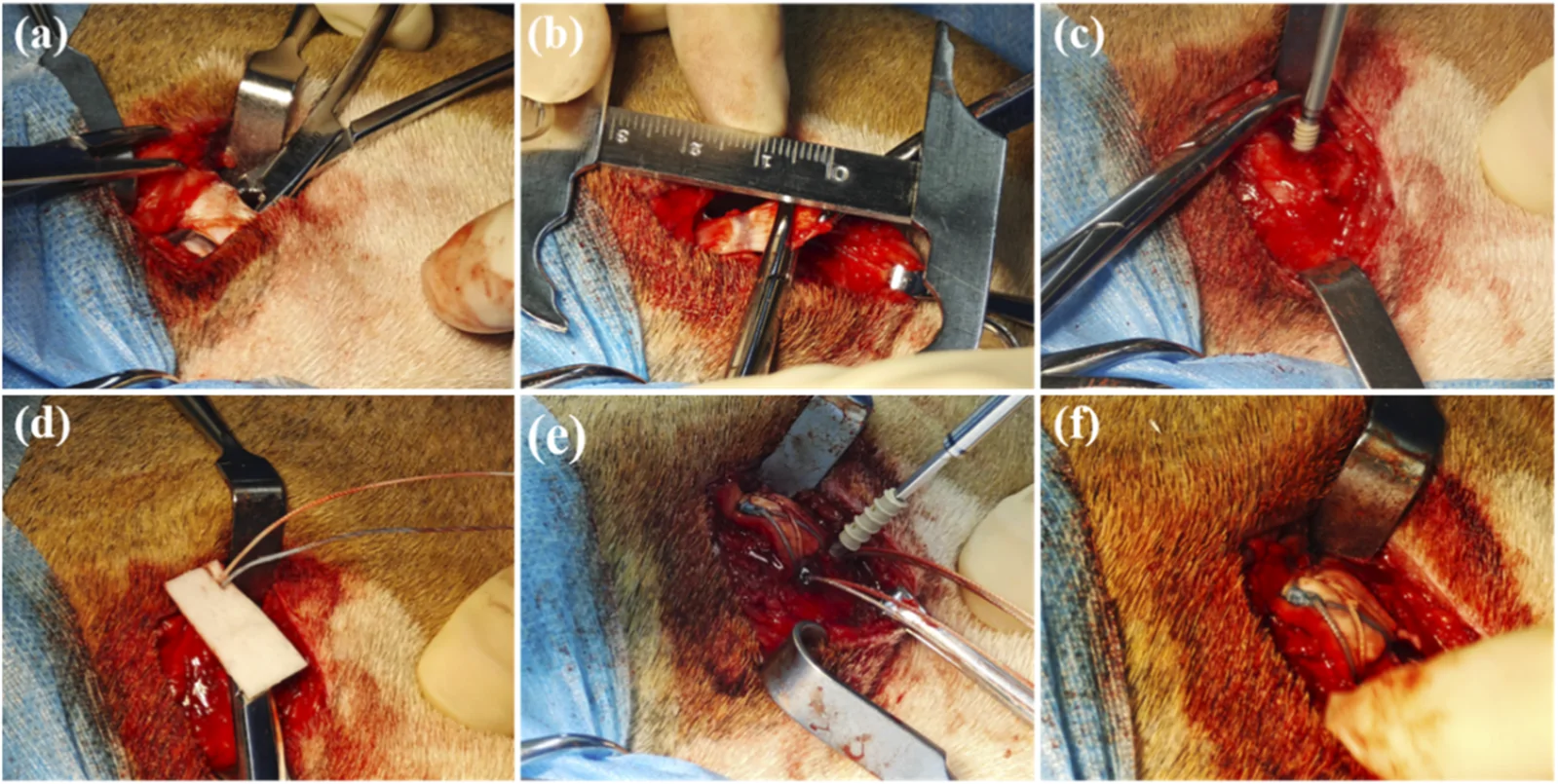
Surgical procedure of patch implantation for rotator cuff injury in a beagle dog, **(a)** Incision and tissue exposure, **(b)** measurement of tendon tissue, **(c)** medial-row anchor placement, **(d)** pADM patch implantation, **(e)** lateral-row anchor placement, and **(f)** final appearance after patch fixation.

### 2.4 Observation and evaluation parameters

Postoperative general health assessments were conducted daily and included evaluations of mental status, dietary intake, activity level, body weight, wound healing, and behavioral performance. Any abnormal findings or postoperative complications, including infection, hematoma, lameness, or other local and systemic adverse reactions, were recorded throughout the observation period.

Venous blood was collected preoperatively and at 2, 4, 6, and 13 weeks postoperatively for complete blood count, blood biochemistry, and coagulation function tests. To further evaluate the systemic inflammatory and immune responses to the implanted material, serum levels of immunoglobulin G (IgG), immunoglobulin M (IgM), and high-sensitivity C-reactive protein (CRP) were measured.

CT scans were performed preoperatively, immediately postoperatively, and at each time point to evaluate anchor position, bone tunnel status, bone resorption, and soft-tissue swelling. MRI was performed immediately postoperatively and at each time point to observe tendon continuity, signal intensity, soft-tissue repair, and joint cavity status.

Tendon thickness was measured on MRI images at three regions of interest (ROIs) preoperatively and at each time point to assess morphological recovery. The three ROIs were defined as follows: Rupture site measured perpendicular to the tendon fibers at the torn edge, 10 mm medial to the medial anchor; Fixation site measured at the patch–tendon interface corresponding to the central axis of the medial anchor; Fixation downstream site measured in the normal tendon region 15 mm lateral to the anchor edge, for comparison with the repaired site.

All measurements were obtained at standardized planes, avoiding tendon folding or stretching, and were performed by a single trained engineer with three repetitions per site; the average was used for analysis. After euthanasia, gross anatomy was performed to examine the morphology, size, color, and texture of major organs (heart, liver, spleen, lung, kidney) and to inspect the operative area, patch condition, and tendon-bone interface.

Tissue from the operative area was fixed, decalcified, dehydrated, and embedded for preparation of pathological sections. Hematoxylin-eosin and Masson staining were performed for evaluation of inflammatory and tissue reactions, semi-quantitative assessment of tendon repair quality using a previously established six-item scoring system (0-3) (Wang and He, 2022), and grading of *in vivo* patch degradation (De Jong et al., 2005).

Intact tendon-humerus specimens were subjected to tensile testing using a universal testing machine (Model E43.104; MTS Industrial Systems (China) Co., Ltd.) equipped with a 0–5 KN load cell. Both ends of each specimen were securely fixed in the grips along the direction of tendon loading, ensuring that the patch-tendon repair interface was precisely centered within the testing fixture without eccentric loading or compressive damage. The test was conducted at a constant loading rate of 30 mm/min (gauge length: 15 mm) for all specimens across all time points and groups. The maximum failure load (N) was determined as the peak point of the load-displacement curve: the ascending phase represents tendon stretching and elastic deformation, the peak represents the maximum load sustained by the repaired specimen, and the subsequent sharp drop indicates failure at the repair interface. All sample preparation and testing procedures were performed by the same operator to ensure consistency. For each time point and group, three parallel specimens were tested, and the average value was used for statistical analysis. The maximum failure load at the tendon-bone junction was recorded to evaluate the mechanical strength of the repaired site.

Blinding was applied as follows: imaging and biomechanical assessments were performed in a single-blind manner, and histopathological evaluations were performed in a double-blind manner (slides re-coded with unblinding after all readings).

### 2.5 Statistical analysis

Data were analyzed with SPSS statistical software. Measurement data are presented as mean ± standard deviation (x̄ ± s). Comparisons between groups were performed using the paired Student’s t-test or the Wilcoxon signed-rank test. Comparisons among different time points were performed using One-Way Analysis of Variance (ANOVA). A P value <0.05 was considered statistically significant. A *post hoc* power analysis was conducted using G*Power 3.1 (α = 0.05) based on tendon thickness data at week 2, where data variability was greatest, representing the worst-case scenario. The achieved power values were 0.987 at the rupture site, 0.931 at the fixation site, and >0.999 at the downstream site, confirming that the sample size (n = 6) provides sufficient statistical power.

## 3 Results

### 3.1 General condition and safety

All dogs survived the postoperative period uneventfully and recovered smoothly. No abnormalities in mental status, diet, respiration, defecation, or spontaneous activity were detected during the observation period, and no adverse events (e.g., death, seizures, infection, hematoma, fat liquefaction, or lameness) occurred. Surgical incisions achieved primary healing without redness, exudation, or ulceration. Body weight showed normal growth trends at all time points, with no abnormal loss or fluctuation.

### 3.2 Hematological and immunological findings

Complete blood count, blood biochemistry, and coagulation parameters remained within normal reference ranges at all postoperative time points (Supplementary Table S2, S3). Although some indices showed statistically significant variations, these were not physiologically significant and did not indicate organ injury, hematopoietic abnormalities, or coagulopathy.

Regarding immune-inflammatory markers (Figure 2), CRP exhibited a transient postoperative elevation, peaking at postoperative day 3 (23.546 ± 17.367 μg/mL) and week 2, then rapidly declining and returning to near-baseline levels by week 4 (5.550 ± 2.358 μg/mL), reflecting a typical acute inflammatory response following trauma. In contrast, IgG and IgM levels showed no abnormal fluctuations at any time point, suggesting that humoral immune function was not significantly affected and that the material did not elicit abnormal immune responses.

**FIGURE 2:**
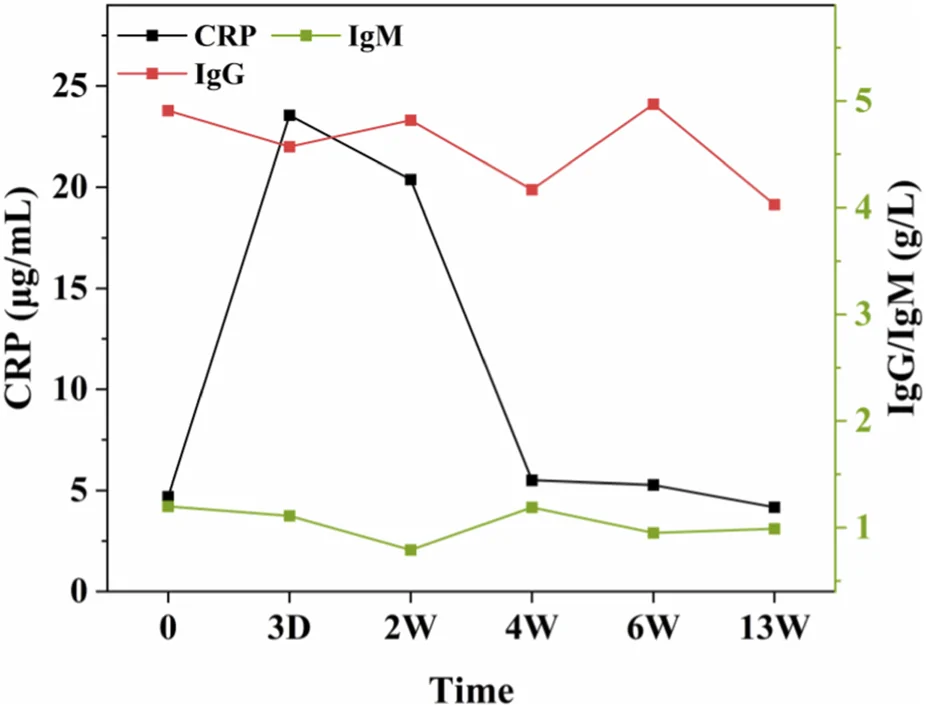
Postoperative serum levels of CRP, IgG, and IgM measured at 3 days, 2, 4, 6 and 13W.

### 3.3 Imaging evaluation

CT examination showed regular morphology of the bone tunnels in the proximal humerus, stable anchor positions without displacement, loosening, or perforation, and no bone resorption or destruction around the bone tunnels (Figure 3). A small amount of gas and soft-tissue swelling was observed in the operative area immediately after surgery; swelling was reduced at 2 weeks, and soft-tissue morphology had largely normalized by 4, 6 and 13 weeks.

**FIGURE 3:**
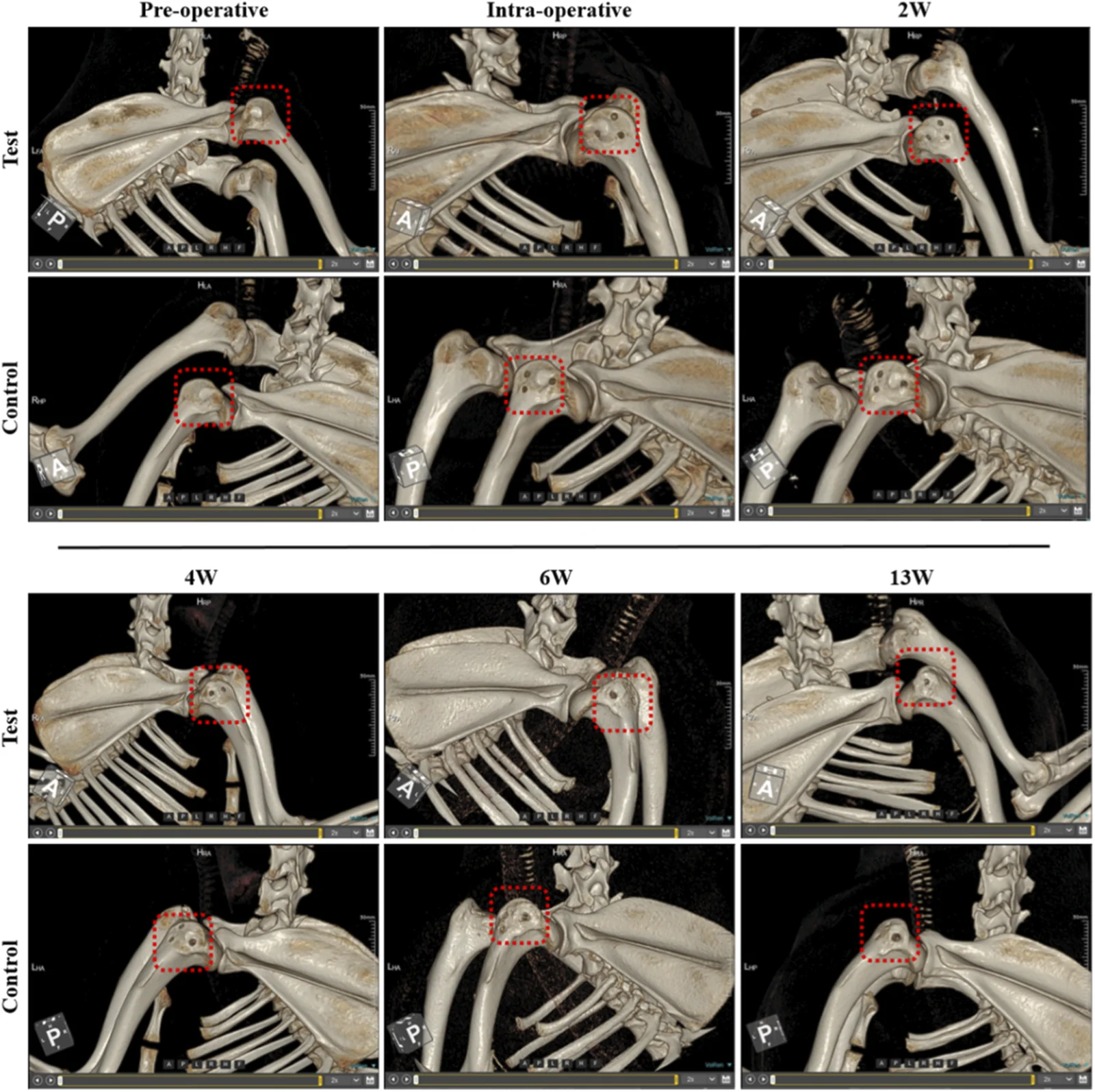
Micro-CT analysis results of test and control group at pre-operative,intra-operative and 2, 4, 6 and 13 weeks. Red box indicates the surgical defect area.

MRI examination revealed discontinuity of the infraspinatus tendon and structural disorganization in the operative area immediately after surgery (Figure 4; Supplementary Figure S1). At 2 weeks, tendon continuity was restored with intermediate signal intensity. At 4 and 6 weeks, tendon signal gradually decreased and structural regularity improved. At 13 weeks, tendon signal was low, and morphology and signal approached those of normal tissue; tendon-bone healing was satisfactory, and no obvious abnormal joint effusion was observed.

**FIGURE 4:**
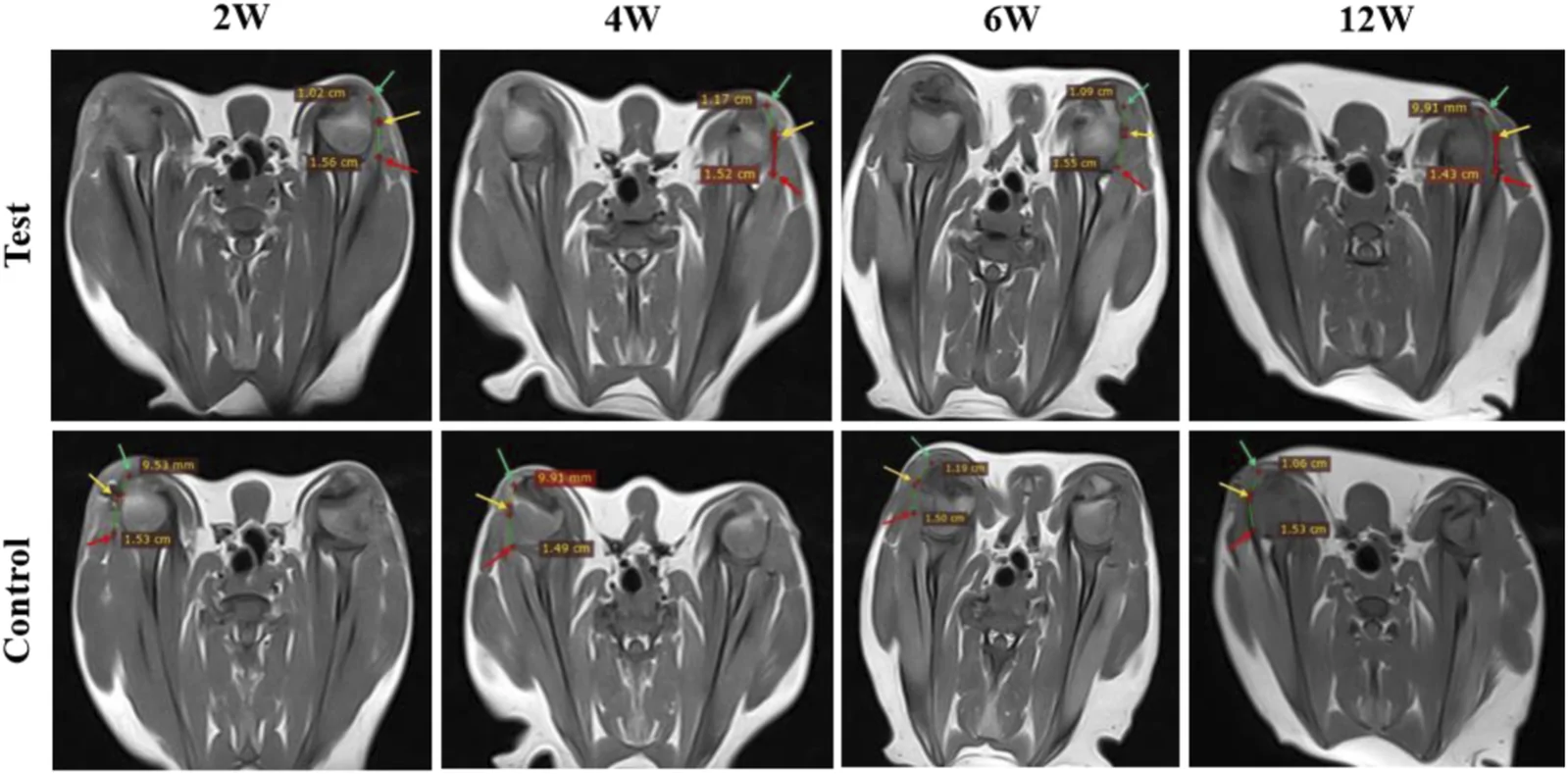
T1 -weighted magnetic resonance imaging images. Red arrow: rupture site (10 mm medial to the medial anchor); yellow arrow: fixation site (central axis of the anchor); green arrow: fixation downstream site (15 mm lateral to the anchor edge).

### 3.4 Changes in tendon thickness

During the early postoperative period (2 and 4 weeks), tendon thickness decreased at all measured sites compared with preoperative values, followed by gradual recovery (Figure 5).

**FIGURE 5:**
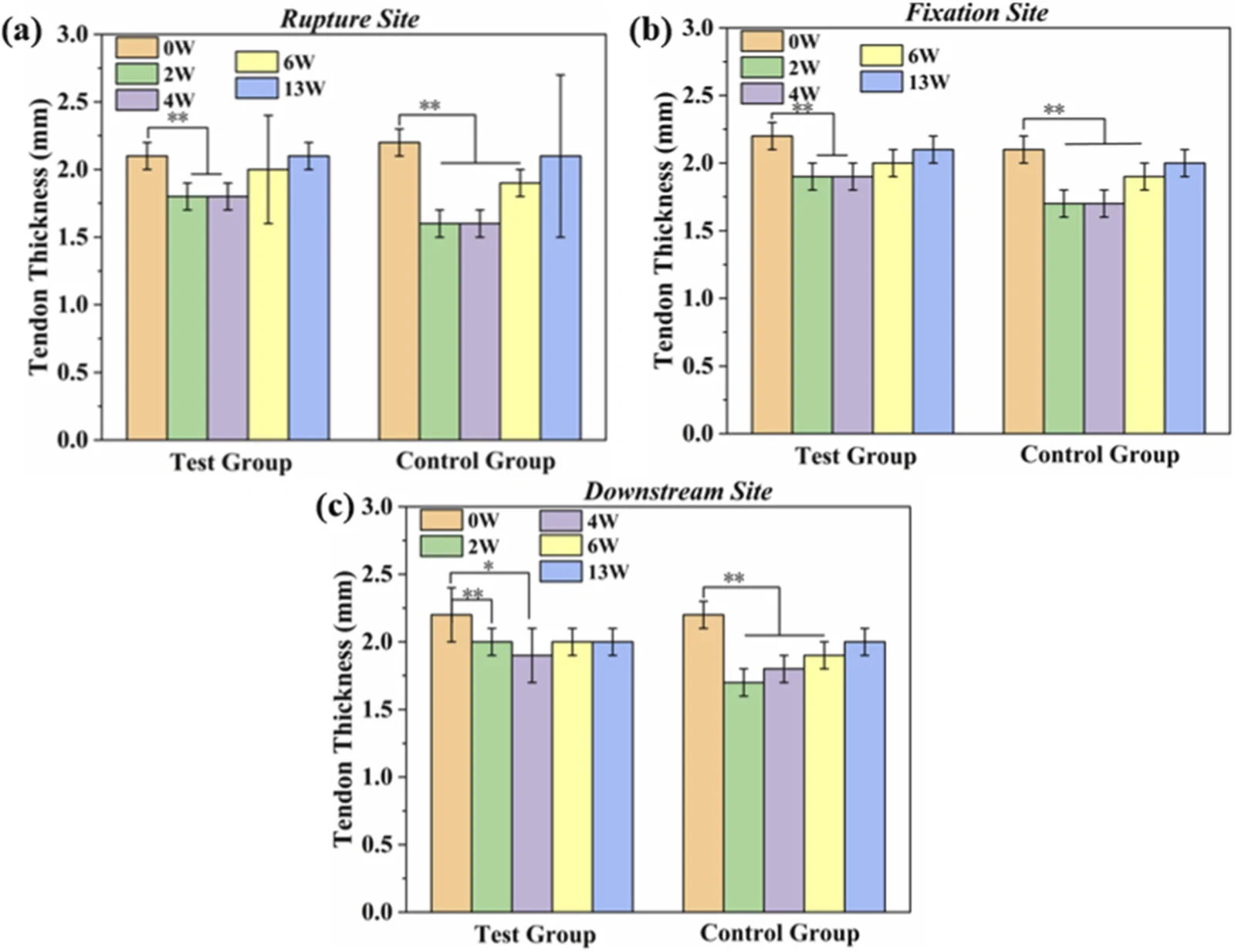
The tendon thickness at the **(a)** rupture, **(b)** fixation and **(c)** fixation downstream site over 13 weeks of postoperatively. Asterisks indicate significant differences using one way ANOVA. (*p < 0.05,**p < 0.01,***p < 0.001).

At the rupture site: Preoperative thickness was 2.2 ± 0.1 mm in the control group and 2.1 ± 0.1 mm in the test group. At 4 weeks, both groups reached their minimum values (control: 1.6 ± 0.1 mm; test: 1.8 ± 0.1 mm). Thereafter, thickness gradually increased, and by 13 weeks, both groups recovered to similar levels (control: 2.1 ± 0.6 mm; test: 2.1 ± 0.1 mm).

At the fixation site: The control group gradually decreased from 2.1 ± 0.1 mm to 1.7 ± 0.1 mm at 4 weeks; while the test group slowly decreased from 2.2 ± 0.1 mm to 1.9 ± 0.1 mm at 4 weeks. The decrease at this site was smaller than that at the stump, and thickness in the test group was slightly higher than in the control group.

Downstream of the fixation site: The control group decreased from 2.2 ± 0.2 mm to 1.8 ± 0.1 mm at 4 weeks; the test group decreased from 2.2 ± 0.2 mm to 1.9 ± 0.2 mm at 4 weeks. At 6 weeks, the test group had recovered to preoperative levels (no significant difference from preoperative values), whereas the control group remained below preoperative levels. At 13 weeks, both groups had recovered to normal levels, with no statistical difference between them.

Overall, tendon thickness in both groups was significantly reduced at 2 and 4 weeks postoperatively compared with preoperative values. At each corresponding time point, the infraspinatus tendon thickness in the test group was consistently greater than that in the control group. At 6 weeks postoperatively, tendon structural recovery in the test group was superior to that in the control group, with near-complete tissue reconstruction observed. By 13 weeks postoperatively, both groups showed restored structural integrity of the infraspinatus tendon.

### 3.5 Gross anatomical observation

Gross anatomy at each time point showed that the major organs (heart, liver, spleen, lung, kidney) were normal in position, shape, color, and texture, with no necrosis, nodules, effusion, or abnormal adhesions. The operative area showed no obvious effusion, suppuration, or tissue necrosis. The patch adhered well to surrounding tissues with a clear interface, and no foreign-body granuloma or abnormal tissue hyperplasia was observed.

### 3.6 Histopathological evaluation

Inflammatory and tissue reaction scores were similar between the test and control groups at all time points, with no statistically significant differences (Table 1; Supplementary Table S4). Both groups exhibited an increase in inflammation after surgery, peaking at 4 weeks (Weighted total score:13) and then gradually declining, which is consistent with the physiological inflammatory process of wound healing.

**TABLE 1:** Histopathological inflammatory reaction scores.

Group	Statistical analysis of inflammation and tissue response			
	2W M(P_25_,P_75_)	4W M(P_25_,P_75_)	6W M(P_25_,P_75_)	13W M(P_25_,P_75_)
Test group	11.00 (11.00,11.50)	13.00 (12.50,13.50)	9.00 (9.00,9.50)	7.00 (6.00,7.50)
Control group	11.00 (11.00,12.00)	13.00 (13.00,13.50)	10.00 (9.50,10.50)	7.00 (7.00,7.50)
Mean of test group	11.33	13.00	9.33	6.67
Mean of control group	11.67	13.33	10.00	7.33
Z	0.258	0.471	0.943	0.471
P	0.796	0.637	0.346	0.637

P < 0.05 indicates a significant difference between the test and control groups, as determined by the Wilcoxon signed-rank test.

As shown in Table 2 and Supplementary Table S5, semi-quantitative scores for tendon repair indicated that the test group had significantly better repair quality at each time point than the control group, as evidenced by more continuous fiber structure, more regular fiber arrangement, nuclear morphology resembling that of normal tendon, milder inflammatory cell infiltration, and more moderate neovascularization.

**TABLE 2:** Semi-quantitative scores for tendon repair.

Group	Statistical analysis of semi-quantitative tendon repair scores			
	2W M(P_25_,P_75_)	4W M(P_25_,P_75_)	6W M(P_25_,P_75_)	13W M(P_25_,P_75_)
Test group	12.00 (11.50,12.00)	8.00 (8.00,8.00)	4.00 (4.00,4.00)	2.00 (1.50,2.00)
Control group	16.00 (15.50,16.50)	10.00 (9.50,10.00)	6.00 (5.50,6.50)	4.00 (3.50,4.50)
Mean of test group	11.67	8.00	4.00	1.67
Mean of control group	16.00	9.67	6.00	4.00
Z	1.993	2.121	2.087	1.993
P	0.046	0.034	0.037	0.046

P < 0.05 indicates a significant difference between the test and control groups, as determined by the Wilcoxon signed-rank test.

Histological evaluation of the tendon-bone interface at serial time points revealed distinct repair patterns between the test and control groups (Figures 6, 7). At 2 weeks, the control group exhibited acute inflammation with abundant polymorphonuclear leukocytes (>30%) and extensive discontinuity of newly formed collagen fibers. The test group showed milder lymphocytic infiltration (inflammatory extent and neovascularization limited to 20–30%), with a structurally intact patch and only slight peripheral dissolution; collagen fibers maintained overall continuity with focal interruptions, and nascent fibers had begun merging with residual tendon-bone interface fibers.

**FIGURE 6:**
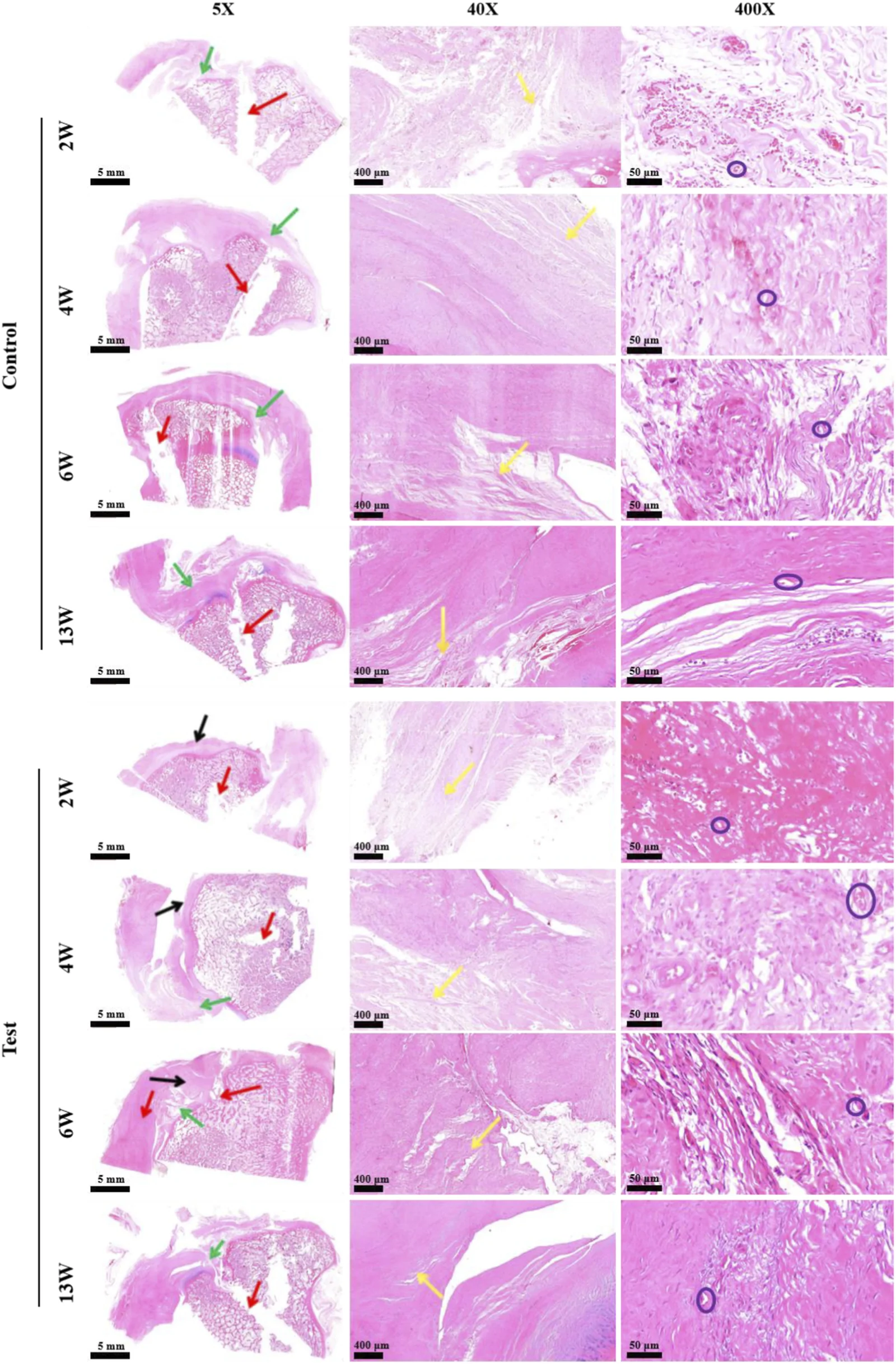
HE staining of control and test group at 2, 4, 6 and 13 weeks. Red arrow: implanted anchor area; black arrow: patch; green arrow: The interface between newly formed fibers and original tendon bones; yellow arrow: newly formed fibers; purple circle: blood vessel.

**FIGURE 7:**
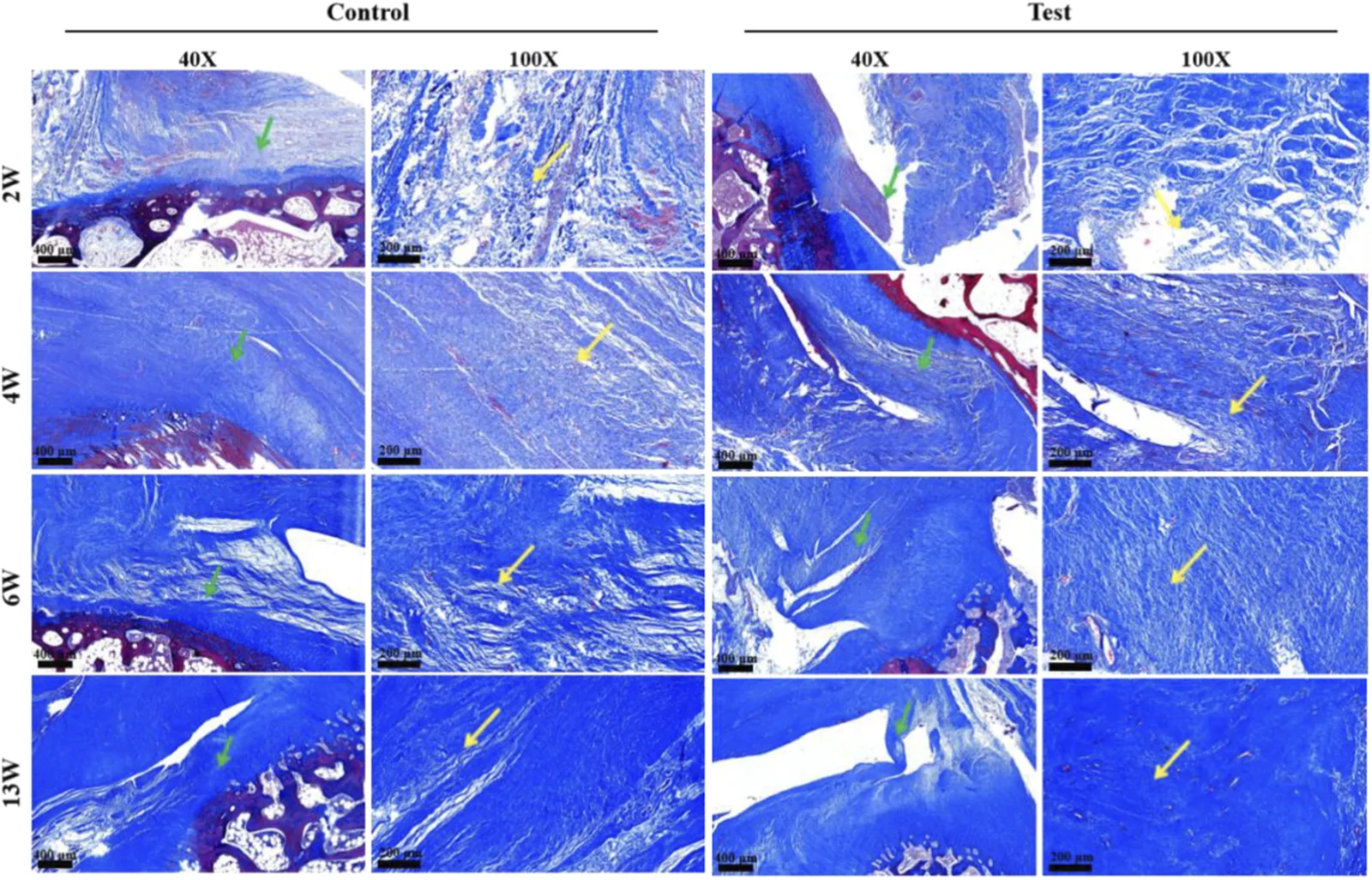
Masson staining of control and test group at 2, 4, 6 and 13 weeks. The green arrow: The interface between newly formed fibers and original tendon bones; yellow arrow: newly formed fibers.

At 4 weeks, the test group transitioned earlier to the proliferative/remodeling phase, with polymorphonuclear and lymphocytic infiltration reduced to minimal levels and macrophages present. Collagen continuity improved markedly, leaving only minor discontinuous areas, while the patch remained intact. The control group still retained some polymorphonuclear leukocytes and showed moderately loose fiber arrangement.

At 6 weeks, the test group demonstrated synchronized degradation-regeneration, with inflammatory infiltration and neovascularization decreasing to 10–19% and cell density normalizing first. The patch showed moderate degradation (cracks/fragments) with host collagen ingrowth. In the control group, although fibers became continuous and mildly loose, macrophage infiltration and neovascularization remained elevated (20–30%), indicating a more active remodeling state.

At 13 weeks, the patch had completely degraded in the test group, replaced by continuous autologous tissue with mature elongated, spindle-shaped nuclei; inflammatory infiltration was <10% and no anchor-site inflammation was observed. The control group displayed continuous fibers but with slightly elevated cell density and more rounded nuclei, suggesting delayed maturation. Extensive fiber-interface integration was evident in both groups.

In summary, the biological patch demonstrated three core advantages: attenuation of early inflammation, controlled degradation with orderly regeneration, and promotion of late physiological maturation. Complete, synchronous replacement by autologous tissue without residue was confirmed histologically, supporting its feasibility and superiority as a functional scaffold for tendon-bone healing.

### 3.7 Biomechanical testing

At 2, 4, 6 and 13 weeks postoperatively, the maximum failure load at the tendon-bone junction was significantly higher in the test group than in the control group (Figure 8). The P-values for 2, 4, 6 and 13 weeks are 0.013, 0.049, 0.014, and 0.036, respectively.

**FIGURE 8:**
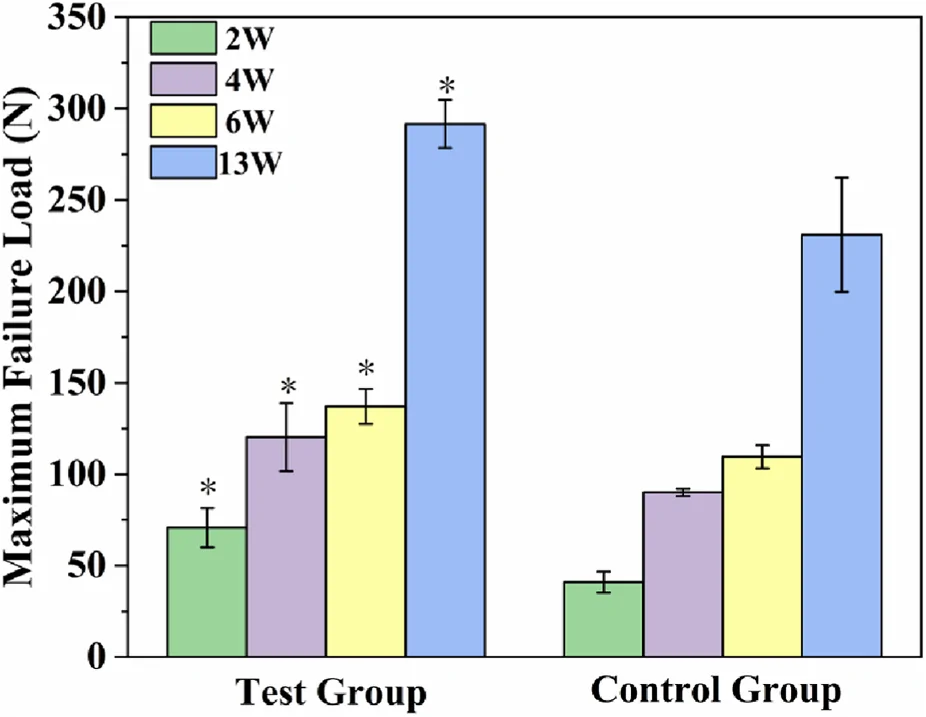
Mechanical testing data of maximum force to failure after 2, 4, 6 and 13W. Asterisks indicate significant differences using t-test between the test group and control group at the same time point (*p < 0.05,**p < 0.01,***p < 0.001).

With increasing postoperative time, mechanical strength gradually improved in both groups, with a more pronounced enhancement in the test group, indicating that the biological patch effectively enhanced the mechanical stability of the repair site and supported early rehabilitation.

## 4 Discussion

The high retear rate following repair of massive rotator cuff tears is attributed to multiple factors, including tendon degeneration, insufficient vascularity, limited biological healing capacity at the tendon-bone interface, and persistent complex mechanical loading (Mcelvany et al., 2015). Conventional suture-only repair restores anatomical continuity to some extent but fails to maintain long-term mechanical stability at the repair interface or effectively promote histological healing of the tendon-bone junction for large or severely degenerative rotator cuff tears (Mandaleson, 2021; Longo et al., 2021). Consequently, biological material-based augmentation has become a major research direction in rotator cuff repair. In the present study, using a beagle dog rotator cuff injury model and a 13-week multi-dimensional observation protocol, we systematically evaluated the safety, degradation behavior, and healing-promoting effects of a pADM patch for rotator cuff augmentation.

Safety is the primary prerequisite for clinical translation of biological patches. In this study, ADM patch implantation did not provoke abnormal inflammatory or immune responses; hematological, biochemical, and immunological indices remained within normal ranges, major organs were grossly intact, and histocompatibility was favorable. Local inflammatory responses in both groups showed an early increase followed by a late decrease, indicating that the ADM patch did not exacerbate the local inflammatory burden. This safety profile has important clinical significance because some commonly used biological materials, for example, SIS patches, despite being widely studied due to their ready availability have been shown to elicit rejection reactions and pronounced inflammation upon early transplantation (Cooper and Mirzayan, 2016; Walton et al., 2007; Toth et al., 2025). In contrast, the ADM patch used in this study demonstrated clear advantages in inflammatory control.

The core advantage of ADM lies in its retention of the three-dimensional ECM network, which provides a scaffold for adhesion, migration, and proliferation of fibroblasts and tenocytes (Brown and Badylak, 2014; Gadre et al., 2024). Compared with synthetic materials, ADM preserves key ECM components including type I and type III collagen, glycosaminoglycans (GAGs), elastin, and various matrix-bound proteins during decellularization (Brown and Badylak, 2014). These components not only maintain microstructural stability but also regulate cell behavior and local regeneration by releasing bioactive signals, thereby promoting orderly collagen deposition and tissue regeneration (Rijal, 2017; Liu et al., 2025). Native ECM materials also possess the ability to modulate the local immune microenvironment. Some studies have indicated that such materials can promote macrophage polarization from the M1 to the M2 phenotype, thereby reducing chronic inflammation and fibrosis risk and enhancing tissue regeneration capacity (Cornelison et al., 2018; Zhang et al., 2022). In the present study, the absence of persistent inflammation and the superior tissue healing observed in the test group indirectly support the hypothesis that the ADM patch may improve the repair environment through such immunomodulatory mechanisms.

During tendon-bone healing, the arrangement and density of collagen fibers are important morphological indicators of tissue maturity (Thomopoulos et al., 2015). Normal tendon tissue exhibits a highly ordered, dense collagen fiber structure aligned along stress directions, whereas early repair tissue typically presents as a disorganized, loose collagen network (Darrieutort-Laffite et al., 2024). As tissue remodeling progresses, collagen fibers gradually realign to form a more continuous, dense, and oriented mature structure (Kanazawa et al., 2016; Sohn and Cho, 2025). Masson staining in the present study showed that the test group had more continuous, dense, and oriented collagen fiber arrangement compared with the control group, suggesting that the ADM patch effectively promotes ECM remodeling and tissue maturation, bringing the repaired tissue closer to normal tendon architecture. A meta-analysis by Hurley et al. of five randomized controlled trials (307 patients) showed that the retear rate in the acellular collagen matrix patch augmentation group was 11% compared with 34% in the control group (Hurley et al., 2024). This clinical evidence quantitatively supports the histological basis of collagen remodeling promoted by ADM patches. These findings are consistent with previous studies, indicating that ADM not only provides passive mechanical support but also actively improves tendon-bone healing quality by regulating ECM reconstruction and the local microenvironment (Flury et al., 2018; Gillespie et al., 2016).

Angiogenesis is another important factor influencing tendon-bone healing (Brown and Badylak, 2014; Liu et al., 2025). Moderate neovascularization provides oxygen, nutrients, and cellular sources to the repair area, facilitating inflammation resolution and tissue regeneration (Zhang et al., 2022; Neishabouri et al., 2022). The native ECM structure retained by ADM serves as a scaffold for endothelial cell migration and microvessel ingrowth, and some matrix degradation products may also exert pro-angiogenic effects (Darrieutort-Laffite et al., 2024). Xu et al. (Xu et al., 2012) implanted a porcine, α-gal-reduced ADM patch in a primate rotator cuff repair model and observed abundant vascular structures at 3 months, with vessel density decreasing to levels similar to normal tendon by 6 months. That study also confirmed no significant inflammation by immunochemical and serum analyses, with histological remodeling into parallel collagen fiber alignment. These data are highly consistent with our observation of “moderate early neovascularization followed by gradual reduction and maturation” in the test group, indicating that ADM contributes to establishing a stable and orderly repair microenvironment.

The match between material degradation behavior and the tissue healing process is a core parameter for evaluating biological patch performance (Liu et al., 2025). An ideal patch should gradually degrade as new tissue matures while providing adequate mechanical support, achieving dynamic replacement. Through multi-time-point histological observation, the present study revealed a progressive degradation pattern of the ADM patch in the beagle rotator cuff repair model: “early structural integrity–midstage degradation with fibrous tissue ingrowth–late complete resorption,” with complete degradation occurring at approximately 13 weeks (Xu et al., 2012). This time course largely coincides with the critical remodeling phase of tendon-bone healing, indicating that the degradation kinetics of the patch are well matched to tissue repair demands. Ide et al. (Ide et al., 2009) in a rat ADM-reconstructed rotator cuff defect model, performed histological and biomechanical evaluations at 2, 6, and 12 weeks postoperatively and found that within 12 weeks the ADM patch had already been completely integrated histologically into a structure resembling normal controls, although collagen alignment remained somewhat irregular. Nicholson et al. (Nicholson et al., 2007) compared the integration behavior of cross-linked pADM patch (Zimmer Collagen Repair Patch) and porcine SIS patch in an ovine rotator cuff repair model. At 9 weeks, most of the SIS material had been resorbed, whereas the ADM patch still had macrophages attached on its surface; by 24 weeks, macrophages had disappeared from the ADM group, and the patch was completely integrated into the surrounding tissue with vascular and fibroblastic invasion (Nicholson et al., 2007). The complete resorption at 13 weeks in the present study may reflect species differences or the effect of patch cross-linking degree on degradation rate.

Biomechanical testing further confirmed that the test group had significantly higher tendon-bone junction strength at all time points, with a particularly prominent advantage in the early postoperative period. This directly reduces the risk of early retear and provides a mechanical basis for more aggressive early rehabilitation, which has clear clinical implications. Combined with histological observations, it can be concluded that the ADM patch achieves sustained improvement in repair performance by enhancing the mechanical integrity of the repair structure, promoting collagen maturation, and facilitating tendon-bone interface integration.

In clinical practice, for rotator cuff tears that are large, degenerative, or of poor soft-tissue quality, suture-only repair often yields unsatisfactory results. The ADM patch can be used as an augmentation layer covering the repair interface to reduce tension, improve the healing environment, and increase repair success rates. This study provides systematic preclinical evidence from a large animal model, comprehensively validating the safety and efficacy of the ADM patch for rotator cuff augmentation and supporting its further clinical translation.

## 5 Conclusion

In this study, we systematically evaluated the safety and efficacy of a pADM patch for rotator cuff augmentation in a beagle dog rotator cuff injury model. The results demonstrated that the patch possesses good biocompatibility and *in vivo* safety, without inducing abnormal inflammatory responses, immune rejection, or major organ toxicity. Regarding tissue healing, the ADM patch significantly promoted tendon structure recovery, accelerated tendon-bone healing, and improved biomechanical strength at the repair site. Moreover, the patch exhibited stepwise degradation characteristics, and its degradation kinetics matched the host tissue remodeling process, with complete resorption by 13 weeks. In summary, the pADM patch demonstrated favorable safety and efficacy in this acute canine rotator cuff injury model, although further studies are warranted to validate its applicability in human chronic degenerative rotator cuff conditions.

### 5.1 Limitations and future directions

This study is a large-animal self-controlled experiment with a limited sample size. The observation period was set to 13 weeks, which, although covering the main healing phase, still requires further investigation of longer-term *in vivo* reactions and long-term safety. The experiment was based on a beagle dog infraspinatus injury model, which has inherent differences from human rotator cuff injury in terms of pathological characteristics, mechanical environment, and healing capacity; thus, extrapolation of the results to clinical practice warrants caution and must be complemented by human studies.

In addition, several limitations of this study should be acknowledged. This study used a healthy, acute rotator cuff injury model, which differs fundamentally from chronic degenerative rotator cuff tears in humans characterised by tendon degeneration, fatty infiltration, muscle atrophy, and impaired intrinsic healing capacity and the present findings may not directly translate to this more clinically representative setting. Functional outcome assessments (e.g., joint range of motion, gait analysis) were not performed, which limits our ability to comprehensively evaluate the functional recovery after repair. The absence of molecular-level evaluations at the tendon-bone interface (e.g., growth factor expression, gene regulatory networks) restricts our understanding of the underlying mechanisms by which pADM promotes tendon-bone healing. Furthermore, this study was conducted in only a single animal species and did not include a head-to-head comparison with other commercially available biological patches, making it difficult to fully assess the superiority of pADM over existing products.

Future research may include multi-center, prospective clinical trials to further evaluate the safety and efficacy of the patch in humans. At the same time, imaging quantitative evaluation, molecular biological assays, and other means should be combined to deeply elucidate the molecular mechanisms by which the decellularized matrix regulates tendon-bone healing, providing a more precise theoretical basis for material optimization and clinical protocol improvement.

## Data Availability

The raw data supporting the conclusions of this article will be made available by the authors, without undue reservation.
