# Supplementary material for: pADM patch augmentation for rotator cuff repair: In vivo safety and efficacy in a canine model
Source: Front Bioeng Biotechnol. 2026 Oct 8;14:1912857. doi: 10.3389/fbioe.2026.1912857 (PMC13687306; doi:10.3389/fbioe.2026.1912857)
Supplement: Supplementary file 1 [file Supplementaryfile1.docx]

**Supplementary materials**

**pADM Patch Augmentation for Rotator Cuff Repair: *In Vivo* Safety and Efficacy in a Canine Model**

*Jianbo Li^a,b^, Qingjun kong^b,*^, Qian Xu^b,*^, Kuansong Wang^a,*^*

*a Department of Pathology, Xiangya Hospital, School of Basic Medical Science, Central South University, 410078, Changsha, Hunan, China*

*b R&D Center, BioPic (Chongqing) Biotechnology Co., Ltd., 401329, Chongqing, Chongqing, China*

***** Corresponding Author:**

Email address:

[qingjun.kong@deltamedical.cn;](mailto:qingjun.kong@deltamedical.cn;) [qian.xu@deltamedical.cn;](mailto:qian.xu@deltamedical.cn;) wangks001@csu.edu.cn

**Table S1 ARRIVE 2.0 checklist**

| **No.** | **Title** | **File/Project ID** |
| --- | --- | --- |
| 1 | Sample Submission Form | GYA25011017A |
| 2 | Protocol | GYA25011017A |
| 3 | IACUC Meeting Minutes | EPINM-F-AW-004-02 |
| 4 | Animal ID Correspondence Record | EPINM--F-BAM-004-01 |
| 5 | Special Husbandry Record | EPINM-F-AM-011-02 |
| 6 | Body Weight Record | EPINM-F-BAM-002-02 |
| 7 | Blood Collection Record | EPINM-F-AT-027-02 |
| 8 | Preclinical Sample Transfer Record | EPINM-F-BAM-017-01 |
| 9 | Blood Test Results | GYA25011017A |
| 10 | Animal Transfer Record | EPINM-F-BAM-020-01 |
| 11 | Medication Administration Record | EPINM-F-BAT-001-02 |
| 12 | Intravenous Catheterization Record | EPINM-F-BAT-003-01 |
| 13 | Anesthesia Record (Large Animal) | EPINM-F-BAT-002-03 |
| 14 | Experimental/Surgical Record | Record Form and Instrument Evaluation Form |
| 15 | Postoperative Clinical Observation Evaluation Criteria | EPINM-F-BAT-055-01 |
| 16 | Postoperative Clinical Observation Record | EPINM-F-BAT-043-02 |
| 17 | Tissue/Specimen Collection Record | EPINM-F-BAT-024-02 |
| 18 | Gross Pathology Examination Record | EPINM-F-BAT-023-02 |
| 19 | Carcass Transfer Record | EPINM-F-AM-010-03 |
| 20 | Report | GYA25011017A-CN |
| Note: Non‑applicable items are marked as “NA”. | | |

**Table S2 Blood biochemistry results in beagle dogs at each time point**

| Period | | TBIL | TP | ALB | TG | ALT | AST | ALP | UREA | CREA | GLU | GGT | TC | P | K | Na | Cl | Ca | pH |
| --- | --- | --- | --- | --- | --- | --- | --- | --- | --- | --- | --- | --- | --- | --- | --- | --- | --- | --- | --- |
|  |  | μmol/L | g/L | g/L | μmol/L | U/L | U/L | U/L | mmol/L | mmol/L | mmol/L | U/L | mmol/L | mmol/L | mmol/L | mmol/L | mmol/L | mmol/L |  |
| Reference range | | 0.09-2.40 | 52.29-82.78 | 22.3-33.5 | 0.11-2.64 | 21.8-116.3 | 17.4-58.6 | 21.2-168.5 | 2.63-9.23 | 35.8-195.0 | 3.18-6.70 | 1.5-8.6 | 2.68-6.85 | 0.94-2.65 | 3.90-5.14 | 137.4-147.0 | 102.0-125.2 | 1.96-2.65 | 7.42-7.79 |
| Pre operative | x̄±SD | 1.01±0.29 | 55.43±2.57 | 25.22±1.74 | 0.59±0.21 | 40.67±7.26 | 31.5±4.31 | 87.8±35.01 | 5.72±1.38 | 48.48±6.22 | 5.75±0.63 | 6.77±6.78 | 4.05±0.86 | 1.67±0.17 | 4.63±0.29 | 140.13±0.8 | 107.7±2.43 | 1.95±0.27 | 7.57±0.04 |
| 2W | x̄±SD | 1.18±0.37 | 60.92±1.51 | 25.83±1.74 | 0.65±0.16 | 65.9±57.06 | 27.63±2.78 | 130.58±46.76 | 4.2±0.65 | 45.42±4.86 | 4.84±0.65 | 6.45±3.94 | 4.6±0.59 | 1.64±0.18 | 4.51±0.13 | 139.13±1.7 | 115.43±2.52 | 1.93±0.09 | 7.58±0.03 |
|  | P value | 0.43 | 0.002* | 0.588 | 0.613 | 0.35 | 0.123 | 0.133 | 0.049* | 0.405 | 0.049* | 0.93 | 0.265 | 0.793 | 0.448 | 0.262 | 0.001  ** | 0.858 | 0.609 |
| Pre operative | x̄±SD | 1.34±0.29 | 60.75±1.48 | 25.98±1.43 | 0.54±0.12 | 59.03±19.71 | 33.35±7.37 | 69.52±38.98 | 3.63±0.74 | 49.42±6.87 | 4.67±0.83 | 4.83±0.87 | 4.21±0.84 | 1.3±0.28 | 4.46±0.11 | 141.78±1.44 | 108.2±2.2 | 2.04±0.11 | 7.57±0.05 |
| 4W | x̄±SD | 1.66±1.08 | 61.45±2.44 | 26.62±1.02 | 0.64±0.08 | 38.63±8.49 | 30.48±5.09 | 81.22±26.97 | 4.13±1.76 | 46.25±9.21 | 4.4±  0.86 | 4.3±  3.4 | 4.95±0.88 | 1.42±0.15 | 4.61±0.22 | 140.9±0.93 | 114.77±9.96 | 2.11±0.17 | 7.61±0.09 |
|  | P value | 0.531 | 0.595 | 0.439 | 0.157 | 0.059 | 0.491 | 0.593 | 0.569 | 0.551 | 0.62 | 0.741 | 0.2 | 0.414 | 0.191 | 0.276 | 0.18 | 0.474 | 0.405 |
| Pre operative | x̄±SD | 1.1±0.23 | 62.12±5.15 | 24.5±1.67 | 0.58±0.24 | 56.63±17.41 | 32.68±3.84 | 100.73±55.72 | 4.19±0.88 | 49.75±1.47 | 4.85±0.71 | 4.68±0.53 | 3.76±0.38 | 1.6±0.21 | 4.66±0.21 | 142.53±0.63 | 107.03±0.97 | 2.24±0.05 | 7.53±0.02 |
| 6W | x̄±SD | 1.36±0.31 | 66.95±4.39 | 27.9±1.26 | 0.67±0.19 | 58.18±12.09 | 31.38±5.61 | 84.72±18.84 | 5.4±0.73 | 55.88±6.11 | 4.91±0.92 | 5.35±1.19 | 4.4±0.44 | 1.32±0.2 | 4.48±0.32 | 143.73±1.87 | 107.17±2.28 | 2.46±0.02 | 7.64±0.05 |
|  | P value | 0.157 | 0.141 | 0.005** | 0.509 | 0.873 | 0.678 | 0.556 | 0.039* | 0.054 | 0.908 | 0.278 | 0.034* | 0.057 | 0.298 | 0.204 | 0.907 | 0.000** | 0.000** |
| Pre operative | x̄±SD | 0.96±0.14 | 61.42±3 | 24.53±1.75 | 0.6±0.17 | 54.57±14.79 | 38.25±9.17 | 64.6±16.71 | 4.97±0.64 | 49±5.95 | 4.71±0.53 | 3.6±2.33 | 3.95±0.94 | 1.56±0.15 | 5.05±0.2 | 148.75±1.29 | 103.93±1.35 | 2.62±0.04 | 7.52±0.03 |
| 13W | x̄±SD | 0.5±0.16 | 62.35±4.64 | 27.47±1.91 | 0.81±0.29 | 41.05±10.43 | 28.13±4.18 | 63.37±16.33 | 5.09±0.9 | 59.22±9.28 | 5.41±0.36 | 3.77±0.71 | 5.53±0.98 | 1.36±0.12 | 4.46±0.22 | 143.08±0.97 | 104.03±1.26 | 2.43±0.05 | 7.58±0.01 |
|  | P value | 0.001** | 0.714 | 0.030* | 0.19 | 0.126 | 0.049* | 0.908 | 0.813 | 0.065 | 0.034 | 0.881 | 0.027* | 0.047 | 0.001** | 0.000  ** | 0.906 | 0.000** | 0.002** |

*The statistically significant difference is marked (*p＜0.05，**p＜0.01，***p＜0.001)*

**Table S3 Complete blood count and coagulation parameters in beagle dogs at each time point**

| Period | | WBC | NEUT | LYMPH | MONO | EO | BASO | RBC | HGB | HCT | MCV | MCH | MCHC | PLT | MPV | APTT | PT | TT | FIB |
| --- | --- | --- | --- | --- | --- | --- | --- | --- | --- | --- | --- | --- | --- | --- | --- | --- | --- | --- | --- |
|  |  | 10⁹/L | 10⁹/L | 10⁹/L | 10⁹/L | 10⁹/L | 10⁹/L | 10¹²/L | g/L | % | fL | pg | g/L | 10⁹/L | fL | s | s | s | g/L |
| Reference range | | 6.08-22.55 | 4.17-18.11 | 0.80-4.96 | 0.26-1.40 | 0.09-1.78 | 0.00-0.07 | 5.50-8.30 | 133-197 | 37.8-55.3 | 62.0-71.8 | 21.7-25.2 | 333-366 | 128-494 | 8.2-13.5 | 9.46-22.43 | 7.07-13.00 | 5.60-20.00 | 0.25-3.00 |
| Pre operative | x̄±SD | 14.38±2.95 | 9.67±1.93 | 3.6±1.22 | 0.72±0.12 | 0.38±0.2 | 0.02±0.01 | 7.09±0.66 | 166.5±18.89 | 49.98±5.14 | 70.5±1.14 | 23.48±0.62 | 332.83±3.98 | 316.67±77.12 | 11.3±1.13 | 11.94±1.87 | 9.44±0.47 | 7.48±0.5 | 2.14±0.46 |
| 2W | x̄±SD | 15.79±3.33 | 11.62±3.12 | 2.88±0.75 | 0.82±0.17 | 0.46±0.12 | 0.02±0.01 | 6.77±0.32 | 157±11.36 | 47.27±2.91 | 69.82±1.79 | 23.18±0.75 | 331.67±4.92 | 358.17±108.71 | 11.3±1.46 | 11.27±0.77 | 7.71±0.74 | 6.9±0.58 | 2.46±0.61 |
|  | P value | 0.497 | 0.263 | 0.29 | 0.298 | 0.496 | 1 | 0.356 | 0.358 | 0.328 | 0.488 | 0.505 | 0.689 | 0.502 | 1 | 0.481 | 0.001** | 0.122 | 0.372 |
| Pre operative | x̄±SD | 14.65±1.51 | 10.71±1.68 | 2.61±0.79 | 0.79±0.12 | 0.48±0.18 | 0.06±0.01 | 7.14±0.71 | 165.83±15.97 | 49.27±4.78 | 69.12±2.78 | 23.23±1.04 | 336.33±3.35 | 369±  82 | 11.2±0.63 | 13.6±2.04 | 10.33±0.9 | 6.53±0.91 | 1.99±0.43 |
| 4W | x̄±SD | 12.38±1.99 | 8.45±1.29 | 2.89±0.87 | 0.58±0.17 | 0.45±0.2 | 0.01±0 | 7.16±0.52 | 168.83±14.14 | 49.68±3.56 | 69.57±3.46 | 23.62±1.38 | 339.33±5.44 | 408.67±98.53 | 10.95±0.78 | 20.95±14.17 | 15.57±5.81 | 6.92±0.88 | 0.87±0.24 |
|  | P value | 0.069 | 0.038* | 0.601 | 0.038* | 0.819 | 0.000** | 0.963 | 0.76 | 0.879 | 0.825 | 0.63 | 0.318 | 0.505 | 0.59 | 0.277 | 0.074 | 0.63 | 0.000** |
| Pre operative | x̄±SD | 17.48±3.98 | 12.61±2.78 | 3.08±0.85 | 0.84±0.27 | 0.91±0.45 | 0.04±0.02 | 6.64±0.36 | 153.67±7.34 | 45.63±2.26 | 68.72±2.01 | 23.17±0.82 | 337.33±2.81 | 435.83±76.01 | 10.2±1.26 | 11.47±0.38 | 12.79±8.98 | 6.61±0.63 | 1.79±0.18 |
| 6W | x̄±SD | 16±2.91 | 11.9±2.17 | 2.91±0.9 | 0.64±0.18 | 0.55±0.2 | 0.01±0.01 | 7.93±0.63 | 183.5±13.26 | 54.13±3.69 | 68.37±1.57 | 23.17±0.6 | 339±3.46 | 376.17±43.06 | 10.05±1.15 | 12.68±0.94 | 15.19±12.78 | 7.41±0.36 | 1.62±0.17 |
|  | P value | 0.518 | 0.664 | 0.763 | 0.195 | 0.13 | 0.008** | 0.003* | 0.001  ** | 0.001** | 0.765 | 1 | 0.423 | 0.158 | 0.848 | 0.023  * | 0.738 | 0.033* | 0.137 |
| Pre operative | x̄±SD | 14.39±3.21 | 10.12±2.44 | 2.87±0.64 | 0.77±0.25 | 0.68±0.37 | 0.03±0.01 | 7.06±0.49 | 159.67±7.99 | 48.23±2 | 68.45±2.49 | 22.68±0.49 | 331.67±5.34 | 223.17±52.53 | 11.28±0.8 | 12.08±1.35 | 9.78±0.71 | 6.49±0.47 | 1.89±0.19 |
| 13W | x̄±SD | 12.13±1.92 | 8.16±1.25 | 2.84±0.56 | 0.6±  0.13 | 0.53±0.29 | 0.01±0 | 7.31±0.54 | 171.67±11.15 | 50.4±3.71 | 68.97±1.82 | 23.48±0.52 | 340.67±4.07 | 260.83±50.98 | 11.68±1.41 | 11.04±1.92 | 8.97±0.97 | 9.94±4.21 | 1.1±0.09 |
|  | P value | 0.207 | 0.141 | 0.936 | 0.205 | 0.723 | 0.000** | 0.46 | 0.079 | 0.277 | 0.715 | 0.032* | 0.013* | 0.277 | 0.594 | 0.347 | 0.163 | 0.098 | 0.000** |

*The statistically significant difference is marked (*p＜0.05，**p＜0.01，***p＜0.001)*

**Fig. S1** T1 and T2-weighted magnetic resonance imaging (MRI) images. The yellow arrow represent the repair area.

**Table S4 Histopathological inflammatory reaction scores**

| **Period** | **Group** | **Inflammatory cell reaction score** | | | | | | **Tissue reaction score** | | | **Weighted total score** | **Degradation score** |
| --- | --- | --- | --- | --- | --- | --- | --- | --- | --- | --- | --- | --- |
|  |  | **Polymorphonuclear leukocytes** | **Lymphocytes** | **Plasma cells** | **Macrophages** | **Giant cells** | **Necrosis** | **Neovascularization** | **Fibrosis** | **Fatty infiltration** |  |  |
| 2W | Test | 2 | 3 | 0 | 0 | 0 | 0 | 1 | 1 | 0 | 12 | 1 |
|  | Test | 2 | 1 | 0 | 1 | 0 | 0 | 2 | 1 | 0 | 11 | 1 |
|  | Test | 1 | 2 | 0 | 1 | 0 | 0 | 2 | 1 | 0 | 11 | 1 |
|  | Control | 3 | 2 | 0 | 0 | 0 | 0 | 2 | 1 | 0 | 13 | NA |
|  | Control | 2 | 1 | 0 | 1 | 0 | 0 | 2 | 1 | 0 | 11 | NA |
|  | Control | 2 | 1 | 0 | 1 | 0 | 0 | 2 | 1 | 0 | 11 | NA |
| 4W | Test | 1 | 1 | 0 | 2 | 0 | 0 | 2 | 2 | 0 | 12 | 1 |
|  | Test | 2 | 1 | 0 | 2 | 0 | 0 | 2 | 2 | 0 | 14 | 2 |
|  | Test | 1 | 2 | 0 | 2 | 0 | 0 | 2 | 1 | 0 | 13 | 3 |
|  | Control | 2 | 2 | 0 | 1 | 0 | 0 | 1 | 2 | 0 | 13 | NA |
|  | Control | 1 | 2 | 0 | 2 | 0 | 0 | 2 | 2 | 0 | 14 | NA |
|  | Control | 2 | 2 | 0 | 1 | 0 | 0 | 1 | 2 | 0 | 13 | NA |
| 6W | Test | 0 | 1 | 0 | 2 | 0 | 0 | 1 | 2 | 0 | 9 | 2 |
|  | Test | 0 | 1 | 0 | 2 | 0 | 0 | 1 | 3 | 0 | 10 | 3 |
|  | Test | 1 | 1 | 0 | 1 | 0 | 0 | 1 | 2 | 0 | 9 | 2 |
|  | Control | 0 | 0 | 0 | 3 | 0 | 0 | 2 | 2 | 0 | 10 | NA |
|  | Control | 0 | 2 | 0 | 1 | 0 | 0 | 2 | 3 | 0 | 11 | NA |
|  | Control | 0 | 1 | 0 | 2 | 0 | 0 | 1 | 2 | 0 | 9 | NA |
| 13W | Test | 0 | 1 | 0 | 1 | 0 | 0 | 1 | 2 | 0 | 7 | 4 |
|  | Test | 0 | 0 | 0 | 2 | 0 | 0 | 1 | 3 | 0 | 8 | 4 |
|  | Test | 0 | 0 | 0 | 1 | 0 | 0 | 1 | 2 | 0 | 5 | 4 |
|  | Control | 0 | 1 | 0 | 1 | 0 | 0 | 2 | 2 | 0 | 8 | NA |
|  | Control | 0 | 1 | 0 | 0 | 0 | 0 | 2 | 3 | 0 | 7 | NA |
|  | Control | 0 | 1 | 0 | 1 | 0 | 0 | 1 | 2 | 0 | 7 | NA |

**Table S5 Semi‑quantitative scores for tendon repair**

| **Period** | **Group** | **Core parameter scores（0-3points per item）** | | | | | | **Total score** |
| --- | --- | --- | --- | --- | --- | --- | --- | --- |
|  |  | **Fiber structure** | **Fiber alignment** | **Round nuclei** | **Inflammation** | **Neovascularization** | **Cell density** |  |
| 2W | Test | 2 | 2 | 2 | 2 | 2 | 1 | 11 |
|  | Test | 2 | 2 | 2 | 2 | 2 | 2 | 12 |
|  | Test | 2 | 2 | 2 | 2 | 2 | 2 | 12 |
|  | Control | 3 | 2 | 3 | 3 | 3 | 3 | 17 |
|  | Control | 2 | 2 | 3 | 3 | 3 | 3 | 16 |
|  | Control | 2 | 2 | 3 | 3 | 3 | 2 | 15 |
| 4W | Test | 1 | 1 | 1 | 2 | 2 | 1 | 8 |
|  | Test | 0 | 1 | 1 | 2 | 2 | 2 | 8 |
|  | Test | 1 | 1 | 1 | 2 | 1 | 2 | 8 |
|  | Control | 0 | 1 | 2 | 2 | 2 | 2 | 9 |
|  | Control | 1 | 2 | 1 | 2 | 2 | 2 | 10 |
|  | Control | 1 | 1 | 1 | 2 | 3 | 2 | 10 |
| 6W | Test | 0 | 1 | 1 | 1 | 1 | 0 | 4 |
|  | Test | 0 | 1 | 1 | 0 | 1 | 1 | 4 |
|  | Test | 0 | 1 | 1 | 0 | 1 | 1 | 4 |
|  | Control | 0 | 1 | 1 | 1 | 2 | 1 | 6 |
|  | Control | 0 | 1 | 1 | 0 | 2 | 1 | 5 |
|  | Control | 1 | 1 | 1 | 1 | 2 | 1 | 7 |
| 13W | Test | 0 | 1 | 1 | 0 | 0 | 0 | 2 |
|  | Test | 0 | 0 | 1 | 0 | 0 | 0 | 1 |
|  | Test | 0 | 1 | 0 | 0 | 1 | 0 | 2 |
|  | Control | 1 | 1 | 1 | 1 | 1 | 0 | 5 |
|  | Control | 0 | 1 | 1 | 0 | 1 | 1 | 4 |
|  | Control | 0 | 1 | 1 | 0 | 0 | 1 | 3 |
