# Supplementary material for: pADM patch augmentation for rotator cuff repair: In vivo safety and efficacy in a canine model
Source: Front Bioeng Biotechnol. 2026 Oct 8;14:1912857. doi: 10.3389/fbioe.2026.1912857 (PMC13687306; doi:10.3389/fbioe.2026.1912857)
Supplement: Supplementary file 2 [file Supplementaryfile2.docx]

**Supplementary materials**

**pADM Patch Augmentation for Rotator Cuff Repair: *In Vivo* Safety and Efficacy in a Canine Model**

*Jianbo Li^a,b^, Qingjun kong^b,*^, Qian Xu^b,*^, Kuansong Wang^a,*^*

*a Department of Pathology, Xiangya Hospital, School of Basic Medical Science, Central South University, 410078, Changsha, Hunan, China*

*b R&D Center, BioPic (Chongqing) Biotechnology Co., Ltd., 401329, Chongqing, Chongqing, China*

***** Corresponding Author:**

Email address:

[qingjun.kong@deltamedical.cn;](mailto:qingjun.kong@deltamedical.cn;) [qian.xu@deltamedical.cn;](mailto:qian.xu@deltamedical.cn;) wangks001@csu.edu.cn

**Table S1 ARRIVE 2.0 Essential 10 checklist**

| **No.** | **Item** | **Content** |
| --- | --- | --- |
| 1 | Study Design | **Study objective:** To evaluate the safety, efficacy, and degradation of the biological patch.  **Study groups:** Test group and control group.  **Experimental unit:** Each animal served as an independent experimental unit. Bilateral shoulder rotator cuff surgeries were performed on each animal, with the left side receiving the test article and the right side receiving the control, to avoid bias due to individual differences.  **Study time points:** Based on the clinical healing time of the intended application site and the degradation characteristics of the product, the observation periods were set at 2 weeks, 4 weeks, 6 weeks, and 13 weeks post‑surgery. |
| 2 | Sample Size | According to the Guidance on Animal Study for Medical Devices – Part II: Study Design, Implementation, and Quality Assurance and following the "3R" principle, this study used a within‑animal (same‑subject) comparison between test and control. Six animals (n=6) were used at each observation time point, totaling 24 animals across four time points. A post‑hoc power analysis (using tendon thickness as a key healing indicator) with α=0.05 (uncorrected) yielded a power >0.8, confirming that the sample size met statistical requirements. |
| 3 | Inclusion/Exclusion Criteria | **Inclusion criteria:** Beagle dogs, age 15~18 months, body weight 13~15 kg; a 7‑day quarantine period with no abnormalities in behavior, activity, food intake, etc.; and normal haematological findings after quarantine.  **Exclusion criteria:** Abnormal pre‑operative physical examination, weight or age not meeting criteria, signs of infection, or abnormal haematological results.  **Criteria for removal:** Death due to non‑experimental factors, severe post‑operative infection, surgical implant failure, withdrawal from the study, or missing/ invalid data. No intraoperative incidents leading to failure or death occurred. |
| 4 | Randomisation | **Randomisation of animal allocation:** After the 7‑day quarantine, animals that passed the quarantine were assigned temporary identification numbers by randomisation. These numbers carried no information about the animals. The project leader used these temporary numbers to allocate animals to different observation time points.  **Strategy to control confounding variables:** All surgeries were performed by the same surgeon, in the same operating room, with anaesthesia strictly following SOPs, and post‑operative housing in the same facility to ensure consistency and control variables. |
| 5 | Blinding | **Animal grouping:** After quarantine, the project leader had only the temporary identification numbers and was blinded to the animals' details when performing random grouping and designing the protocol. Image reading and mechanical testing were both blinded: Biomechanical tests were performed by technicians who were unaware of group allocation and intervention details, with the same engineer conducting all recordings.  **Histopathological reading used double‑blinding:** pathology technicians re‑labelled slides with pure numbers or letter combinations to completely mask animal group, dose, and other information, ensuring that pathologists could not identify the slides' origin. After all slides were read blindly, the pathologists submitted their diagnostic reports to the non‑clinical study director for data analysis and report writing (unblinding). |
| 6 | Outcome Assessment | 1. **General observation:** Daily post‑operative observations were made for each animal, including general appearance, mental state, behaviour, food intake, and faecal characteristics. The surgical site was also inspected for local tissue reactions, haematoma, effusion, or infection. Body weight was measured before surgery and at 2, 4, 6, and 13 weeks after surgery. 2. **Haematology evaluation:** Blood biochemistry, haematology (complete blood count), and coagulation function were assessed before surgery and at 2, 4, 6, and 13 weeks post‑surgery. Changes in each parameter over time were evaluated within the same animal.   **3. Immunological evaluation:** Blood samples (3 mL) were collected before surgery and at 3 days, 2 weeks, 4 weeks, 6 weeks, and 13 weeks after surgery for determination of IgG, IgM, and high‑sensitivity C‑reactive protein (hs‑CRP). Changes in these markers over time were evaluated within the same animal.  **4. CT examination:** CT scans were performed before surgery, immediately after surgery, and at 2, 4, 6, and 13 weeks post‑surgery. Pre‑operative CT was used to localise the surgical site; immediate post‑operative CT confirmed anchor placement; and subsequent scans at 2, 4, 6, and 13 weeks (before necropsy) re‑confirmed anchor status.  **5. MRI examination:** MRI of the bilateral surgical sites was performed immediately after surgery and at 2, 4, 6, and 13 weeks to evaluate soft‑tissue healing and inflammatory response.  **6. Tendon thickness measurement:** Tendon thickness was measured before surgery, immediately after surgery, and at 2, 4, 6, and 13 weeks. Measurements were taken at three different locations on the tendon (3 mm distal to the fixation site), with three repeated measurements per location, to analyse changes in tendon thickness and assess recovery.  **7. Gross observation:** At each time point (2, 4, 6, and 13 weeks), animals were euthanised, and major organs (heart, liver, spleen, lungs, kidneys) were inspected visually and photographed for position, size, colour, texture, and any abnormalities such as effusion or necrosis. If visible lesions were found, tissue samples were taken for histopathology; if no lesions were visible, no samples were collected. The surgical site was also inspected and photographed for wound healing and changes in the implant, including tissue integration and morphological changes.  **8. Histopathology:** At each time point (2, 4, 6, and 13 weeks), the surgical site and surrounding tissues were harvested intact. After anchor removal, specimens were decalcified and processed for routine histopathology. HE staining was performed to evaluate the effect on surrounding tissues (e.g., inflammatory response); Masson staining was used to assess collagen formation and degradation. The right‑side (control) specimens were processed identically.  **9. Tissue biomechanics:** At each time point (2, 4, 6, and 13 weeks), the tendon‑bone complex (tendon and humerus) was harvested. Biomechanical testing was performed using a testing machine, with the fixture angle adjusted to align with the anatomical direction of the tendon, ensuring that the tensile load acted along the tendon‑bone interface and avoiding shear forces. The test was conducted at room temperature 25±2°C, humidity 50‑70%, with the tendon surface kept moist using saline‑soaked gauze. The specimens were pulled at 30 mm/min until failure. The maximum failure load was recorded to evaluate the biomechanical strength of tendon‑bone healing. |
| 7 | Statistical Methods | Haematology data, tendon thickness measurements, and biomechanical data were analysed using SPSS 26.0 with t‑tests for comparison. Histopathological inflammation/tissue reaction scores and semi‑quantitative tendon repair scores were analysed using Wilcoxon rank‑sum tests. |
| 8 | Experimental Animals | 1. **Animal use permit:** Scope of Application \| Conventional environment (applicable to rabbits, guinea pigs, hamsters, sheep, minipigs, dogs, monkeys) Barrier environment (SPF: mice, rats, guinea pigs, rabbits, hamsters); Animal use license No. \| SYXK (Gui) 2023‑0005 2. **Animal information:** Species \| Beagle dogs; Grade \| Conventional; Number/sex \| 28 animals, male/female half and half; Weight \| 11‑16 kg; Supplier \| Hunan Hengxinwang Biotechnology Co., Ltd; Animal production license \| SCXK (Xiang) 2024‑0010; Animal quality certificate No. \| 430792251100008542 3. **Animal housing and management:** Housing density \| Single cage (cage size: 1120 mm × 1000 mm × 2080 mm ); Cage equipment \| Stainless steel double‑tier; Identification \| Microchip implanted in the neck and cage cards (with study number, individual ID, entry date, remarks); Quarantine period \| At least 7 days; Feed \| Laboratory dog maintenance diet (compliant with GB 14924.1, GB 13078, GB 14924.2); Water \| Free access via automatic drinkers (drinking water meets GB 14925‑2023) 4. **Animal room environmental control:** Temperature \| 16‑28°C; Relative humidity \| 30‑70% RH; Air changes \| ≥8 times/h; Lighting \| 2h light/12h dark cycle, full‑spectrum fluorescent lamps |
| 9 | Experimental Procedures | 1. **Animal and instrument preparation:** 1) Animals arrived at the laboratory and were acclimatised for 7 days before the study. Food and water were withheld for 8 hours before surgery. 2) Surgical instruments and consumables were prepared according to requirements. 3) Multi‑parameter monitors and anaesthesia machines were set up and tested. 4) Pre‑operative body weight was recorded for each animal. 5) Appropriate sizes of test and control devices were selected based on body weight. 2. **Anaesthesia and monitoring:** 1) Propofol was administered intravenously for induction, and the animal was placed prone on the operating table. 2) Endotracheal intubation was performed, and the animal was positioned in left lateral recumbency. 3) Anaesthesia was maintained with isoflurane via a ventilator. 4) Central venous pressure and oxygen saturation were monitored. 5) Heart rate, blood pressure, body temperature, and oxygen saturation were continuously monitored throughout surgery using a multi‑parameter monitor. 3. **Surgical site preparation and disinfection:** 1) Both shoulders were shaved, initially disinfected with 75% alcohol, and dried. 2) The area was further disinfected with iodine, and sterile drapes were placed. 4. **Sample pre‑treatment:** The biological patch was rehydrated in normal saline for 30 minutes. 5. **Surgical procedure:** 1) Rotator cuff injury model: With the animal in right lateral recumbency, a skin incision was made centered on the lower edge of the left scapular spine. The skin and subcutaneous tissue were incised, and surrounding muscles were bluntly dissected to expose the insertion of the infraspinatus tendon on the humeral head. The distal tendon was cut and elevated from its attachment at the greater tubercle, and a 5 mm × 5 mm segment was resected. Tendon thickness was measured. 2) Repair of the rotator cuff defect: The soft tissue over the footprint was scraped off. A 3.2 mm drill was used to create a tunnel approximately 5.0 mm lateral to the attachment, and one 3.4 mm × 14 mm suture anchor was gently inserted. The tendon was sutured with a suture passer. The patch was trimmed to 18 mm × 10 mm and sutured to the tendon. Two 3.5 mm × 16 mm lateral row anchors were implanted using a 3.3 mm drill at the greater tubercle, and the patch was secured with a suture‑bridge technique. The patch was further reinforced on both sides, and the incision was closed in layers. 3) The animal was repositioned to left lateral recumbency, and the same procedure was performed on the right shoulder to create the defect and repair it using the same method without the patch (control). 6. **Post‑operative care:** Meloxicam and ceftiofur sodium were given subcutaneously for 5 consecutive days to provide analgesia and prevent infection. If wound infection or exudation occurred, the wound was cleaned and disinfected with iodine. The veterinarian could administer additional interventions as needed based on recovery and animal welfare. |
| 10 | Results | Post‑operatively, all animals showed normal general condition, behaviour, and surgical sites. Body weight remained stable throughout the study. Haematology, biochemistry, coagulation, and immunological parameters showed no abnormal responses. Gross examination of organs revealed no lesions or abnormalities. Combined with clinical observations, the product demonstrated a satisfactory safety profile. CT and MRI scans showed no abnormal changes at the suture anchor fixation sites, no abnormal bone resorption, and the infraspinatus muscle gradually recovered over time, with complete recovery by 13 weeks. At 2 and 4 weeks, tendon thickness in both the test (left) and control (right) groups was significantly lower than pre‑operative values; however, the test group had greater infraspinatus thickness than the control. By 6 weeks, tendon repair in the test group was superior to the control, and the test group had essentially completed repair. The test group showed faster repair than the control group. At 2 weeks, the biological patch still possessed some tensile strength. At 4, 6, and 13 weeks, the patch was encased in fibrous tissue and undergoing degradation, making it impossible to retrieve for tensile strength evaluation. At all time points (2, 4, 6, and 13 weeks), the tissue biomechanical strength of the test group was significantly higher than that of the control group, indicating better infraspinatus repair. Inflammatory and tissue reaction scores showed no significant differences between the test and control groups at any time point. The tendon repair scores, however, were significantly different between the two groups at all time points, with the test group showing better repair, indicating that the biological patch promoted infraspinatus tendon healing and had completely degraded by 13 weeks post‑surgery. In conclusion, under the conditions of this study, the biological patch demonstrated no safety concerns during rotator cuff repair. The test product was superior to the control in repairing the infraspinatus injury model. |

**Table S2 Blood biochemistry results in beagle dogs at each time point**

| Period | | TBIL | TP | ALB | TG | ALT | AST | ALP | UREA | CREA | GLU | GGT | TC | P | K | Na | Cl | Ca | pH |
| --- | --- | --- | --- | --- | --- | --- | --- | --- | --- | --- | --- | --- | --- | --- | --- | --- | --- | --- | --- |
|  |  | μmol/L | g/L | g/L | μmol/L | U/L | U/L | U/L | mmol/L | mmol/L | mmol/L | U/L | mmol/L | mmol/L | mmol/L | mmol/L | mmol/L | mmol/L |  |
| Reference range | | 0.09-2.40 | 52.29-82.78 | 22.3-33.5 | 0.11-2.64 | 21.8-116.3 | 17.4-58.6 | 21.2-168.5 | 2.63-9.23 | 35.8-195.0 | 3.18-6.70 | 1.5-8.6 | 2.68-6.85 | 0.94-2.65 | 3.90-5.14 | 137.4-147.0 | 102.0-125.2 | 1.96-2.65 | 7.42-7.79 |
| Pre operative | x̄±SD | 1.01±0.29 | 55.43±2.57 | 25.22±1.74 | 0.59±0.21 | 40.67±7.26 | 31.5±4.31 | 87.8±35.01 | 5.72±1.38 | 48.48±6.22 | 5.75±0.63 | 6.77±6.78 | 4.05±0.86 | 1.67±0.17 | 4.63±0.29 | 140.13±0.8 | 107.7±2.43 | 1.95±0.27 | 7.57±0.04 |
| 2W | x̄±SD | 1.18±0.37 | 60.92±1.51 | 25.83±1.74 | 0.65±0.16 | 65.9±57.06 | 27.63±2.78 | 130.58±46.76 | 4.2±0.65 | 45.42±4.86 | 4.84±0.65 | 6.45±3.94 | 4.6±0.59 | 1.64±0.18 | 4.51±0.13 | 139.13±1.7 | 115.43±2.52 | 1.93±0.09 | 7.58±0.03 |
|  | P value | 0.43 | 0.002* | 0.588 | 0.613 | 0.35 | 0.123 | 0.133 | 0.049* | 0.405 | 0.049* | 0.93 | 0.265 | 0.793 | 0.448 | 0.262 | 0.001  ** | 0.858 | 0.609 |
| Pre operative | x̄±SD | 1.34±0.29 | 60.75±1.48 | 25.98±1.43 | 0.54±0.12 | 59.03±19.71 | 33.35±7.37 | 69.52±38.98 | 3.63±0.74 | 49.42±6.87 | 4.67±0.83 | 4.83±0.87 | 4.21±0.84 | 1.3±0.28 | 4.46±0.11 | 141.78±1.44 | 108.2±2.2 | 2.04±0.11 | 7.57±0.05 |
| 4W | x̄±SD | 1.66±1.08 | 61.45±2.44 | 26.62±1.02 | 0.64±0.08 | 38.63±8.49 | 30.48±5.09 | 81.22±26.97 | 4.13±1.76 | 46.25±9.21 | 4.4±  0.86 | 4.3±  3.4 | 4.95±0.88 | 1.42±0.15 | 4.61±0.22 | 140.9±0.93 | 114.77±9.96 | 2.11±0.17 | 7.61±0.09 |
|  | P value | 0.531 | 0.595 | 0.439 | 0.157 | 0.059 | 0.491 | 0.593 | 0.569 | 0.551 | 0.62 | 0.741 | 0.2 | 0.414 | 0.191 | 0.276 | 0.18 | 0.474 | 0.405 |
| Pre operative | x̄±SD | 1.1±0.23 | 62.12±5.15 | 24.5±1.67 | 0.58±0.24 | 56.63±17.41 | 32.68±3.84 | 100.73±55.72 | 4.19±0.88 | 49.75±1.47 | 4.85±0.71 | 4.68±0.53 | 3.76±0.38 | 1.6±0.21 | 4.66±0.21 | 142.53±0.63 | 107.03±0.97 | 2.24±0.05 | 7.53±0.02 |
| 6W | x̄±SD | 1.36±0.31 | 66.95±4.39 | 27.9±1.26 | 0.67±0.19 | 58.18±12.09 | 31.38±5.61 | 84.72±18.84 | 5.4±0.73 | 55.88±6.11 | 4.91±0.92 | 5.35±1.19 | 4.4±0.44 | 1.32±0.2 | 4.48±0.32 | 143.73±1.87 | 107.17±2.28 | 2.46±0.02 | 7.64±0.05 |
|  | P value | 0.157 | 0.141 | 0.005** | 0.509 | 0.873 | 0.678 | 0.556 | 0.039* | 0.054 | 0.908 | 0.278 | 0.034* | 0.057 | 0.298 | 0.204 | 0.907 | 0.000** | 0.000** |
| Pre operative | x̄±SD | 0.96±0.14 | 61.42±3 | 24.53±1.75 | 0.6±0.17 | 54.57±14.79 | 38.25±9.17 | 64.6±16.71 | 4.97±0.64 | 49±5.95 | 4.71±0.53 | 3.6±2.33 | 3.95±0.94 | 1.56±0.15 | 5.05±0.2 | 148.75±1.29 | 103.93±1.35 | 2.62±0.04 | 7.52±0.03 |
| 13W | x̄±SD | 0.5±0.16 | 62.35±4.64 | 27.47±1.91 | 0.81±0.29 | 41.05±10.43 | 28.13±4.18 | 63.37±16.33 | 5.09±0.9 | 59.22±9.28 | 5.41±0.36 | 3.77±0.71 | 5.53±0.98 | 1.36±0.12 | 4.46±0.22 | 143.08±0.97 | 104.03±1.26 | 2.43±0.05 | 7.58±0.01 |
|  | P value | 0.001** | 0.714 | 0.030* | 0.19 | 0.126 | 0.049* | 0.908 | 0.813 | 0.065 | 0.034 | 0.881 | 0.027* | 0.047 | 0.001** | 0.000  ** | 0.906 | 0.000** | 0.002** |

*The statistically significant difference is marked using t-test (*p＜0.05，**p＜0.01)*

**Table S3 Complete blood count and coagulation parameters in beagle dogs at each time point**

| Period | | WBC | NEUT | LYMPH | MONO | EO | BASO | RBC | HGB | HCT | MCV | MCH | MCHC | PLT | MPV | APTT | PT | TT | FIB |
| --- | --- | --- | --- | --- | --- | --- | --- | --- | --- | --- | --- | --- | --- | --- | --- | --- | --- | --- | --- |
|  |  | 10⁹/L | 10⁹/L | 10⁹/L | 10⁹/L | 10⁹/L | 10⁹/L | 10¹²/L | g/L | % | fL | pg | g/L | 10⁹/L | fL | s | s | s | g/L |
| Reference range | | 6.08-22.55 | 4.17-18.11 | 0.80-4.96 | 0.26-1.40 | 0.09-1.78 | 0.00-0.07 | 5.50-8.30 | 133-197 | 37.8-55.3 | 62.0-71.8 | 21.7-25.2 | 333-366 | 128-494 | 8.2-13.5 | 9.46-22.43 | 7.07-13.00 | 5.60-20.00 | 0.25-3.00 |
| Pre operative | x̄±SD | 14.38±2.95 | 9.67±1.93 | 3.6±1.22 | 0.72±0.12 | 0.38±0.2 | 0.02±0.01 | 7.09±0.66 | 166.5±18.89 | 49.98±5.14 | 70.5±1.14 | 23.48±0.62 | 332.83±3.98 | 316.67±77.12 | 11.3±1.13 | 11.94±1.87 | 9.44±0.47 | 7.48±0.5 | 2.14±0.46 |
| 2W | x̄±SD | 15.79±3.33 | 11.62±3.12 | 2.88±0.75 | 0.82±0.17 | 0.46±0.12 | 0.02±0.01 | 6.77±0.32 | 157±11.36 | 47.27±2.91 | 69.82±1.79 | 23.18±0.75 | 331.67±4.92 | 358.17±108.71 | 11.3±1.46 | 11.27±0.77 | 7.71±0.74 | 6.9±0.58 | 2.46±0.61 |
|  | P value | 0.497 | 0.263 | 0.29 | 0.298 | 0.496 | 1 | 0.356 | 0.358 | 0.328 | 0.488 | 0.505 | 0.689 | 0.502 | 1 | 0.481 | 0.001** | 0.122 | 0.372 |
| Pre operative | x̄±SD | 14.65±1.51 | 10.71±1.68 | 2.61±0.79 | 0.79±0.12 | 0.48±0.18 | 0.06±0.01 | 7.14±0.71 | 165.83±15.97 | 49.27±4.78 | 69.12±2.78 | 23.23±1.04 | 336.33±3.35 | 369±  82 | 11.2±0.63 | 13.6±2.04 | 10.33±0.9 | 6.53±0.91 | 1.99±0.43 |
| 4W | x̄±SD | 12.38±1.99 | 8.45±1.29 | 2.89±0.87 | 0.58±0.17 | 0.45±0.2 | 0.01±0 | 7.16±0.52 | 168.83±14.14 | 49.68±3.56 | 69.57±3.46 | 23.62±1.38 | 339.33±5.44 | 408.67±98.53 | 10.95±0.78 | 20.95±14.17 | 15.57±5.81 | 6.92±0.88 | 0.87±0.24 |
|  | P value | 0.069 | 0.038* | 0.601 | 0.038* | 0.819 | 0.000** | 0.963 | 0.76 | 0.879 | 0.825 | 0.63 | 0.318 | 0.505 | 0.59 | 0.277 | 0.074 | 0.63 | 0.000** |
| Pre operative | x̄±SD | 17.48±3.98 | 12.61±2.78 | 3.08±0.85 | 0.84±0.27 | 0.91±0.45 | 0.04±0.02 | 6.64±0.36 | 153.67±7.34 | 45.63±2.26 | 68.72±2.01 | 23.17±0.82 | 337.33±2.81 | 435.83±76.01 | 10.2±1.26 | 11.47±0.38 | 12.79±8.98 | 6.61±0.63 | 1.79±0.18 |
| 6W | x̄±SD | 16±2.91 | 11.9±2.17 | 2.91±0.9 | 0.64±0.18 | 0.55±0.2 | 0.01±0.01 | 7.93±0.63 | 183.5±13.26 | 54.13±3.69 | 68.37±1.57 | 23.17±0.6 | 339±3.46 | 376.17±43.06 | 10.05±1.15 | 12.68±0.94 | 15.19±12.78 | 7.41±0.36 | 1.62±0.17 |
|  | P value | 0.518 | 0.664 | 0.763 | 0.195 | 0.13 | 0.008** | 0.003* | 0.001  ** | 0.001** | 0.765 | 1 | 0.423 | 0.158 | 0.848 | 0.023  * | 0.738 | 0.033* | 0.137 |
| Pre operative | x̄±SD | 14.39±3.21 | 10.12±2.44 | 2.87±0.64 | 0.77±0.25 | 0.68±0.37 | 0.03±0.01 | 7.06±0.49 | 159.67±7.99 | 48.23±2 | 68.45±2.49 | 22.68±0.49 | 331.67±5.34 | 223.17±52.53 | 11.28±0.8 | 12.08±1.35 | 9.78±0.71 | 6.49±0.47 | 1.89±0.19 |
| 13W | x̄±SD | 12.13±1.92 | 8.16±1.25 | 2.84±0.56 | 0.6±  0.13 | 0.53±0.29 | 0.01±0 | 7.31±0.54 | 171.67±11.15 | 50.4±3.71 | 68.97±1.82 | 23.48±0.52 | 340.67±4.07 | 260.83±50.98 | 11.68±1.41 | 11.04±1.92 | 8.97±0.97 | 9.94±4.21 | 1.1±0.09 |
|  | P value | 0.207 | 0.141 | 0.936 | 0.205 | 0.723 | 0.000** | 0.46 | 0.079 | 0.277 | 0.715 | 0.032* | 0.013* | 0.277 | 0.594 | 0.347 | 0.163 | 0.098 | 0.000** |

*The statistically significant difference is marked using t-test (*p＜0.05，**p＜0.01)*

**Fig. S1** T1 and T2-weighted magnetic resonance imaging (MRI) images. The yellow arrow represent the repair area.

**Table S4 Histopathological inflammatory reaction scores**

| **Period** | **Group** | **Inflammatory cell reaction score** | | | | | | **Tissue reaction score** | | | **Weighted total score** | **Degradation score** |
| --- | --- | --- | --- | --- | --- | --- | --- | --- | --- | --- | --- | --- |
|  |  | **Polymorphonuclear leukocytes** | **Lymphocytes** | **Plasma cells** | **Macrophages** | **Giant cells** | **Necrosis** | **Neovascularization** | **Fibrosis** | **Fatty infiltration** |  |  |
| 2W | Test | 2 | 3 | 0 | 0 | 0 | 0 | 1 | 1 | 0 | 12 | 1 |
|  | Test | 2 | 1 | 0 | 1 | 0 | 0 | 2 | 1 | 0 | 11 | 1 |
|  | Test | 1 | 2 | 0 | 1 | 0 | 0 | 2 | 1 | 0 | 11 | 1 |
|  | Control | 3 | 2 | 0 | 0 | 0 | 0 | 2 | 1 | 0 | 13 | NA |
|  | Control | 2 | 1 | 0 | 1 | 0 | 0 | 2 | 1 | 0 | 11 | NA |
|  | Control | 2 | 1 | 0 | 1 | 0 | 0 | 2 | 1 | 0 | 11 | NA |
| 4W | Test | 1 | 1 | 0 | 2 | 0 | 0 | 2 | 2 | 0 | 12 | 1 |
|  | Test | 2 | 1 | 0 | 2 | 0 | 0 | 2 | 2 | 0 | 14 | 2 |
|  | Test | 1 | 2 | 0 | 2 | 0 | 0 | 2 | 1 | 0 | 13 | 3 |
|  | Control | 2 | 2 | 0 | 1 | 0 | 0 | 1 | 2 | 0 | 13 | NA |
|  | Control | 1 | 2 | 0 | 2 | 0 | 0 | 2 | 2 | 0 | 14 | NA |
|  | Control | 2 | 2 | 0 | 1 | 0 | 0 | 1 | 2 | 0 | 13 | NA |
| 6W | Test | 0 | 1 | 0 | 2 | 0 | 0 | 1 | 2 | 0 | 9 | 2 |
|  | Test | 0 | 1 | 0 | 2 | 0 | 0 | 1 | 3 | 0 | 10 | 3 |
|  | Test | 1 | 1 | 0 | 1 | 0 | 0 | 1 | 2 | 0 | 9 | 2 |
|  | Control | 0 | 0 | 0 | 3 | 0 | 0 | 2 | 2 | 0 | 10 | NA |
|  | Control | 0 | 2 | 0 | 1 | 0 | 0 | 2 | 3 | 0 | 11 | NA |
|  | Control | 0 | 1 | 0 | 2 | 0 | 0 | 1 | 2 | 0 | 9 | NA |
| 13W | Test | 0 | 1 | 0 | 1 | 0 | 0 | 1 | 2 | 0 | 7 | 4 |
|  | Test | 0 | 0 | 0 | 2 | 0 | 0 | 1 | 3 | 0 | 8 | 4 |
|  | Test | 0 | 0 | 0 | 1 | 0 | 0 | 1 | 2 | 0 | 5 | 4 |
|  | Control | 0 | 1 | 0 | 1 | 0 | 0 | 2 | 2 | 0 | 8 | NA |
|  | Control | 0 | 1 | 0 | 0 | 0 | 0 | 2 | 3 | 0 | 7 | NA |
|  | Control | 0 | 1 | 0 | 1 | 0 | 0 | 1 | 2 | 0 | 7 | NA |

**Table S5 Semi‑quantitative scores for tendon repair**

| **Period** | **Group** | **Core parameter scores（0-3points per item）** | | | | | | **Total score** |
| --- | --- | --- | --- | --- | --- | --- | --- | --- |
|  |  | **Fiber structure** | **Fiber alignment** | **Round nuclei** | **Inflammation** | **Neovascularization** | **Cell density** |  |
| 2W | Test | 2 | 2 | 2 | 2 | 2 | 1 | 11 |
|  | Test | 2 | 2 | 2 | 2 | 2 | 2 | 12 |
|  | Test | 2 | 2 | 2 | 2 | 2 | 2 | 12 |
|  | Control | 3 | 2 | 3 | 3 | 3 | 3 | 17 |
|  | Control | 2 | 2 | 3 | 3 | 3 | 3 | 16 |
|  | Control | 2 | 2 | 3 | 3 | 3 | 2 | 15 |
| 4W | Test | 1 | 1 | 1 | 2 | 2 | 1 | 8 |
|  | Test | 0 | 1 | 1 | 2 | 2 | 2 | 8 |
|  | Test | 1 | 1 | 1 | 2 | 1 | 2 | 8 |
|  | Control | 0 | 1 | 2 | 2 | 2 | 2 | 9 |
|  | Control | 1 | 2 | 1 | 2 | 2 | 2 | 10 |
|  | Control | 1 | 1 | 1 | 2 | 3 | 2 | 10 |
| 6W | Test | 0 | 1 | 1 | 1 | 1 | 0 | 4 |
|  | Test | 0 | 1 | 1 | 0 | 1 | 1 | 4 |
|  | Test | 0 | 1 | 1 | 0 | 1 | 1 | 4 |
|  | Control | 0 | 1 | 1 | 1 | 2 | 1 | 6 |
|  | Control | 0 | 1 | 1 | 0 | 2 | 1 | 5 |
|  | Control | 1 | 1 | 1 | 1 | 2 | 1 | 7 |
| 13W | Test | 0 | 1 | 1 | 0 | 0 | 0 | 2 |
|  | Test | 0 | 0 | 1 | 0 | 0 | 0 | 1 |
|  | Test | 0 | 1 | 0 | 0 | 1 | 0 | 2 |
|  | Control | 1 | 1 | 1 | 1 | 1 | 0 | 5 |
|  | Control | 0 | 1 | 1 | 0 | 1 | 1 | 4 |
|  | Control | 0 | 1 | 1 | 0 | 0 | 1 | 3 |
